# Recommendations for Interventions to Improve Function in Patients With Lung Cancer: A Clinical Practice Guideline

**DOI:** 10.1002/cam4.70626

**Published:** 2025-07-04

**Authors:** Mary Vargo, Lynn H. Gerber, Laura S. Gilchrist, Mary Insana Fisher

**Affiliations:** ^1^ MetroHealth Rehabilitation Institute Case Western Reserve University Cleveland Ohio USA; ^2^ George Mason University Fairfax Virginia USA; ^3^ St. Catherine University St. Paul Minnesota USA; ^4^ University of Dayton Dayton Ohio USA

**Keywords:** function, mobility, oncology, physical activity, prehabilitation, rehabilitation, survivorship, systematic review

## Abstract

**Introduction:**

Individuals with lung cancer frequently experience functional limitations, yet best practice to achieve functional recovery has not been synthesized. This Clinical Practice Guideline generates evidence‐based recommendations to improve functional outcomes in this population.

**Methods:**

A multispecialty expert workgroup completed a systematic review of the literature from 2010 to 2021 examining interventions for improving patient function at any stage or phase of disease. National Institutes of Health Quality Assessment standards were employed for bias assessment. Recommendations were generated per GRADE methodology, for mobility, physical activity, general function, and social function outcomes, during phases of prehabilitation, surgical post‐operative acute (hospitalization), during‐or‐immediate post‐treatment (first year), and survivorship.

**Results:**

Fifty‐four studies were included. Combined exercise approaches should be used to improve mobility during the Prehabilitation and the During and Immediate Post‐Treatment phases. For physical activity, combined interventions may be beneficial during the Surgical Post‐Operative Acute phase, and multimodal interventions with exercise and education may improve function in the During and Immediate Post‐Treatment phase. Combined exercise may improve general function in the During and Immediate Post‐Treatment phase.

**Conclusion:**

Current evidence emphasizes mobility outcomes, in prehabilitation and early post‐treatment phases, with moderate level benefits of combined aerobic with resistance and/or breathing exercise. Further study is needed into sustainability of performing the interventions and durability of outcomes; increased breadth of interventions and functional outcome domains examined; and exploration of specific contexts including advanced disease, survivorship, high medical complexity and frailty, and caregiver‐related factors. These recommendations are applicable for clinicians including oncologists, rehabilitation specialists, surgeons, primary and pulmonary care providers, nurses, and other supportive care personnel.

## Abstract 2

For lung cancer patients, combined exercise interventions should be performed to improve function and to minimize treatment‐related functional declines, with favorable outcomes seen in both pretreatment and post‐treatment phases, and with mobility outcomes demonstrating the most abundant available evidence. Evidence remains insufficient regarding the impact of unidimensional exercise interventions as well as the effects of non‐physical activity–based interventions on patient function.

## 1 Introduction

Lung cancer is one of the most common cancers in adults and is associated with significant functional limitations. Often diagnosed in people aged 65+, the estimated number of new lung cancer cases in the United States in 2022 was 236,740, with 654,620 individuals in the United States being survivors of lung cancer [1]. Individuals with lung cancer report high rates of disability even at the time of diagnosis [2], lower workforce participation [3], and worse physical function as compared to those with other common cancer types [4].

Overall, the 5‐year survival for lung cancer remains low at 22% yet has improved from 13% in the early 1990s [1], attribute to both improved diagnostic measures and better treatment options [1]. Multimodal treatment is often given, with surgery, radiation, chemotherapy, and immunotherapy as possible treatment options. Recent improvements in surgical techniques, immunotherapies, and possibly rehabilitation have helped to drive the increase in survival rates. Thirty percent of individuals are diagnosed with Stage I disease, a cohort with a current 5‐year survival rate of 65% [1]. Unfortunately, Stage IV disease remains the most common stage at diagnosis, at 37% of presentations [1]. Many survivors are impacted by geriatric‐related comorbidities as well as chronic respiratory conditions, which reduce function, increase care needs, and potentially make them ineligible for treatments associated with high toxicity [5]. While much of the lung cancer rehabilitation literature centers around the post‐surgical treatment phase, a significant percentage of lung cancer patients are not treated surgically, with > 55% of Stages I and II patients receiving surgery, but only 21% of Stage III patients [1]. Altogether these data suggest a heterogeneity of outcomes for this disease and a need to improve rehabilitation of cancer‐related sequelae including functional limitations.

Clinical practice guidelines (CPGs) can be important contributors to selecting appropriate health care interventions. CPGs, when done carefully and without bias, help establish quality medical care and reduce practice variation [6]. In an effort to assure quality and reliability, several organizations have published standards for their development to provide trustworthy recommendations [6, 7, 8, 9]. A quality guideline requires a well‐planned review of the literature with clearly defined criteria that identifies which articles are included and excluded from consideration. Included literature is then reviewed for risk of bias. Lastly, the evidence is synthesized and recommendations for practice are formulated using a system by which the strength of those recommendations is evident [8, 10, 11].

### 1.1 CPGs in Cancer Rehabilitation

Cancer rehabilitation is a process that provides people with cancer diagnoses an assessment of physical or cognitive impairments resulting from the impacts of cancer and its treatment and from comorbid conditions. Physical function encompasses a range of actions including basic physical movements, essential self‐care, and the more complex abilities needed for essential life roles and discretionary activities [12]. Another important contributor to function includes participation, including social, vocational, or family activities. The goals of cancer rehabilitation are to promote and restore function or prevent functional decline during all stages of cancer and survivorship.

In the field of cancer rehabilitation, where there are relatively few specialists, CPGs provide educational value to practitioners, patients, and caregivers/family, and may help extend practice in this under‐sourced area. Additionally, in this field, clinical cohorts may be small, patients' courses may be unique, and large controlled trials may not be feasible to perform. Hence, aggregated literature, properly vetted, can provide useful and reliable treatment guidance [6]. A specific objective of this CPG was to focus on literature examining rehabilitation's impact on the functional impairments in those with lung cancer. In 2016, an NIH‐sponsored Subject Matter Expert Group on cancer rehabilitation published recommendations that included a call to “develop practice guidelines regarding functional assessment, screening for physical impairments, and rehabilitation interventions to enhance the selection of rehabilitation interventions, referrals, and outcomes measurement” [13]. Since then, a systematic review of rehabilitation and exercise guidelines in oncology found high‐quality guidelines related to breast cancer, prostate cancer, head and neck cancer, lung cancer, and thyroid cancer [14]. However, relatively few CPGs examine the impact of specific interventions on physical function and general functional outcomes (i.e., participation) for patients with cancer.

Regarding lung cancer specifically, many systematic reviews and meta‐analyses [15, 16, 17, 18, 19, 20, 21, 22, 23, 24, 25, 26, 27, 28, 29, 30, 31, 32, 33, 34, 35, 36, 37, 38, 39, 40, 41, 42, 43, 44, 45, 46, 47, 48, 49, 50, 51, 52, 53, 54] have been published regarding rehabilitation and related topics (including prehabilitation, activity, and exercise studies) for this population, but clinical practice guidelines have been limited. Stout et al.'s [14] 2021 systematic review of rehabilitation and exercise recommendations in oncology guidelines noted only one Category A guideline (defined as containing recommendations for specific rehabilitation assessments and interventions) with respect to lung cancer meeting AGREE II criteria, which focused on treatment for cough [55]. Another Category A guideline, by Brunelli et al. [56], comprehensively examined fitness for radical treatment of lung cancer but was assessed as below AGREE II threshold. The Brunelli practice guideline heavily focused on preoperative testing as well as on physiological effects of lung cancer treatments in various specific clinical contexts, but its attention to interventions for global function and performance was limited. The current CPG was undertaken to evaluate the impact of rehabilitation interventions on functional outcomes for people with lung cancer, and to synthesize recommendations supported by evidence from clinical trials and/or observational studies. Therefore, the purpose of this CPG is to make recommendations to improve function through rehabilitation of individuals with lung cancer.

## 2 Methods

### 2.1 Background Definitions and Inclusion/Exclusion Criteria

Strict criteria for “function” were chosen for this CPG based on the International Classification of Function [57] which addresses function on the level of anatomy/body part, the whole person, and the whole person in his/her environment. Inclusion criteria required studies to use rehabilitation outcomes that addressed function on the whole‐body level (e.g., mobility and self‐care) or on the level of environmental interaction (social participation). Studies also were required to demonstrate that an intervention was provided or prescribed to improve function, maximize independence, or increase physical activity. Exclusion criteria included research designs such as cohort studies, non‐intervention studies, systematic reviews, or meta‐analyses, studies in non‐lung cancer populations, or single case studies.

While the authors of this CPG acknowledge that anyone with a current or past diagnosis of cancer is a survivor [58], for the purposes of structuring the available evidence, we divided our review in time periods with the following phases of care:

- Prehabilitation—the period from diagnosis up to surgical or medical intervention aimed at treating the lung cancer.
- Surgical Post‐operative Acute—the period of inpatient hospitalization acutely after surgery for lung cancer.
- During and Immediate Post‐Treatment—the period encompassing the active medical management of the individual with cancer, including chemotherapy and radiation treatment, and the healing period following all medical and surgical treatments for cancer, inclusive of the year following oncologic treatments.
- Survivorship—at least 1 year post diagnosis and after medical treatment is completed.

### 2.2 Literature Search

The following bibliographic databases were searched for the period from January 1, 2010 through December 31, 2021: Medline (Ovid), Cochrane Library Databases (Cochrane Library, Wiley), PsycINFO (Ovid), Embase, CINAHL Complete (EBSCO), and Web of Science (Clarivate). The full Medline search strategy is shown (Supplemental Content—Appendix S1). All dedicated lung cancer studies from the original systematic review databank of Sleight et al. [59] from 2010 to 2018 were available to us and were reviewed. A new search spanning January 2019 to December 2021 updated the literature available. While studies from the Sleight et al. [59] review were preselected as focused on function, the additional studies were assessed for inclusion of functional outcome measures and interventions meeting the stringent criteria described above. Search terms for the updated search were the same as per Sleight et al. [59], though narrowed toward lung cancer, and employed the assistance of a professional librarian. References from systematic reviews were manually reviewed for completeness of the literature search. Any articles that emerged incidentally as a downstream effect of the literature search, such as being found in the references of existing articles, could be included.

### 2.3 Study Selection Process

Covidence (Melbourne, Australia) was used to organize the screening and full‐text review process. Titles and abstracts of articles were screened by two independent investigators based on the prespecified inclusion and exclusion criteria of clinical trials of rehabilitation interventions in a lung cancer population with a functional outcome measure. As noted, the intent was to focus rigorously on function. Therefore, studies with symptom or impairment‐level outcomes such as fatigue, dyspnea, pain, muscle strength, pulmonary function studies, or maximum aerobic capacity were not included, even though many such parameters have correlations with function. General health outcomes such as medical complications of care, hospitalization length of stay, or hospitalization rates were also not included. Studies with mixed diagnoses were included only if the outcomes for the lung cancer subgroup were distinctly reported or if the percentage of lung cancer patients constituted 75% or more of the total sample. Full texts of potential included articles were then retrieved and re‐screened to assure criteria were met. Full‐text articles were reviewed independently by two researchers and when uncertainty or conflicting opinions about inclusion existed, articles were brought to the group for consensus.

### 2.4 Study Quality

The National Institutes of Health (NIH) Quality Assessment tool standards [60] were used to assess each included study for risk of bias. Studies were first classified as controlled intervention study, pre‐post treatment study, observational cohort or cross‐sectional study, case control study, or case series and then the appropriate quality review checklist was utilized. The quality assessment criteria for each study design vary slightly in accordance with expected standards for each study type. For example, controlled intervention studies are rated according to randomization, blinding, dropout rates, adherence, similarities between groups in baseline characteristics and background treatments, sample size determined by power analysis, validity of outcome measures, and use of intention‐to‐treat analysis. Only prospective studies were included in the clinical trial and pre/post study categories. Retrospective studies were considered observational and classified as either case controlled or cohort studies [61]. For each study, primary and secondary reviewers independently completed the NIH Quality Assessment worksheet for the article type. The two reviewers then compared their responses for agreement about the type of study and for the individual quality parameters. Upon completion of this process, a quality rating for each article was determined as good, fair, or poor. If significant discrepancies or uncertainties were present for any of these steps, a third reviewer was added, and when necessary, resolved by the full group. A detailed Quality Assessment electronic tracking sheet of the quality information for each paper was maintained. Particular attention was paid to the level of adherence to the intervention in the study population. Adherence was empirically considered at a good level if values of 70% or greater were reported [61, 62, 63, 64, 65].

### 2.5 Data Extraction

The data extracted included: citation, national origin of the study, reviewers, phase of care, tumor stage, setting, final study quality rating, level of evidence, subject numbers—total and analyzed, primary aim, outcome tool for the primary aim, results of the primary aim, secondary aim, outcome tool for the secondary aim, results of the secondary aim (repeating this for any additional secondary aims), cancer treatment, rehabilitation treatment, supervision, intensity, frequency, duration, follow‐up length, control intervention, intensity, frequency, duration, follow‐up length, risks/contraindications to rehabilitation, safety observations, and methodological considerations. The phase of the study was categorized as Prehabilitation, Surgical Post‐Operative Acute, During and Immediate Post‐Treatment, and Survivorship per the operational definitions above. Setting was categorized as hospital, clinic, research lab, community, home‐based, or unspecified. It was also noted whether the authors of the included papers evaluated the extent of their findings being clinically meaningful [66]. If two manuscripts were obviously reporting from the same dataset and demonstrated largely overlapping or redundant information, the two papers were combined into one line item on the data extraction grid. Studies which were designed as feasibility studies were examined but were not included in the final product unless the methodology permitted sufficient conclusions to be drawn regarding functional outcomes.

The level of evidence was indicated as 1 through 5 based on Oxford guidelines [4, 66], and adjusted when appropriate for individual study quality factors; the final level of evidence rating was based on study design and quality rating. For example, a randomized controlled study (RCT) rated as good or fair in the quality analysis phase was assigned an evidence rating of 2; a pre/post study rated as good or fair would be assigned a level of 3. Studies rated as poor on the NIH quality assessment tool were automatically downgraded an evidence level. Studies rated as fair could also be downgraded a level at the discretion of the reviewers; on the other hand, an exemplary RCT with high effect could be assigned an evidence level of 1. Studies that incorporated the highest evidence were included in the final group of articles from which recommendations were generated. Reviewed studies meeting the identified inclusion standards, but which methodologically were rated as “poor” upon bias assessment were not included in the final group of articles. Similarly, studies with levels of evidence of 4 or 5 were not included. However, for the poor quality or lower evidence level (4 or 5) studies, an exception could be made if a unique topic was addressed, not examined in the more highly rated studies.

### 2.6 Outcome Measures

Outcomes were categorized into the following:

- Mobility—basic walking and movement capabilities.
- Physical activity—daily physical activity performed.
- General function—physical and/or self‐care function.
- Social function—family, work, school, leisure, community activities.

Both patient‐reported outcome measures and objectively measured data were included. Note was made of the primary outcome measure of each study, regardless of whether it was a functional measure or not. When a primary aim was not explicitly stated, and multiple outcomes were evaluated, the outcome measure most meaningful to function was assigned as the primary aim. In studies with no explicitly stated primary aim, which assessed an objectively measured outcome, such as six‐minute walk test (6MWT) or timed up and go, the objective measure would be given priority over self‐reported outcome measures, though all function‐related outcome data were noted and included in the analysis. Quality of life (QOL) instruments were included in the analysis within the general function outcome category if relevant functional subscales were also reported; social functioning subscales of global QOL instruments could also be included within the Social Function category. However, the physical activity outcome category did not include physical QOL subscales but focused on measured or self‐reported activity levels.

When possible, clinical meaningfulness was considered if statistical significance was achieved in a study. Clinically meaningful changes were determined based on published minimally clinically important difference (MCID) values, or minimally important difference (MID) values if no MCID was available for each measure. If an outcome measure had no MCID or MID established for a lung cancer population, then values established in pulmonary disease or heart disease populations were used (in that order of preference). The value selected for each outcome measure and the patient population used for identifying important change can be found in Supplemental Content—Appendix S2 [68, 69, 70, 71, 72, 73, 74, 75, 76, 77]. Each rehabilitation clinical trial or study result was reviewed for meeting the MCID or MID metric, and if the study result met the clinically important metric, the outcome was bolded in Tables 1, 2, 3, 4 (Functional Outcomes) and informed the narrative analysis.

**TABLE 1:** Mobility outcomes.

Author, year (level)	Prehab	Surgical post‐op acute	During or immediate post‐treatment	Survivor‐ship	Aerobic	Strength	Breathing	Other intervention	Mobility impact (see key below)
*Prehabilitation*									
Finley 2021 [81] (3)	X				X				NS 6MWT (S)
Licker 2017 [85] (2), Bhatia 2019 [80] (2)	X				X	X			+ **66 m 6MWT** (S)
Morano 2014 [88] (2)	X				X	X		Stress management, nutrition, stretching	NS 6MWT (S)
Lai, Huang, Yang 2017 [83] (2)	X				X		X		+ 19.2 m 6MWT (S)
Lai, Su, Qui 2017 [84] (2)	X				X		X		+ 18.7 m 6MWT (S)
Ma 2021 [136] (2)	X				X		X		+ Combined exercise; 22 m 6MWT; NS for breathing exercise alone (12 m) ++ **49 m 6MWT** combined exercise at day of hospital discharge; 23 m breathing ex; all decreased from preop at day of hospital discharge (P)
Pehlivan 2019 [89] (3)	X				X		X		**+ 53.5 m 6MWT** (N/A; pre/post study)
Huang 2017 [82] (2)	X				X	X	X		**+ 32.7 m 6MWT** combined exercise group; NS in breathing exercise alone group (S)
Minnella 2021 [87] (3)	X				X	X	X	Nutrition, coping	+ **30 m 6MWT** (S vs. N/A; feasibility pre/post study)
Liu 2020 [86] (2)	X				X	X	X	Whey supplement; mental relaxation	**+**, **++ 60.9 m 6MWT** (30 days) (P)
*Prehabilitation + post‐op*									
Pehlivan 2011 [92] (2)	X	X			X		X		+ Max walking distance in intervention group, NS between groups (S)
van der Leeden 2019 [95] (3)	X	X			X		X		− Pre/post test: 6MWT and sit‐to‐stand declined significantly, no long‐term data (S)
*Prehabilitation + during/immediately post treatment*									
Ferreira 2021 [90] (2)	X		X		X	X		Education, nutrition, address anxiety	NS 6MWT for prehab + rehab vs. rehab groups. Sub‐analyses of “unfit” prehab patients showed **+ 34.6 m** in prehab phase, and of “fit” patients in rehab group **+ 28 m** between 4 and 8 weeks post‐op. Both groups meet or exceed baseline performance by 8 weeks post‐op (P)
Sommer 2016 [93] (2)	X		X		X	X	X	Health promotion and counseling	Prehab not feasible, limited participation + 16 m 6MWT (S)
*Prehabilitation + post‐op + during/immediately post treatment*									
Lafaro 2020 [91] (3)	X	X	X		X	X			Indeterminate for prehab (feasibility study; only one pre‐op time point) + 1.8 SPPB NS for 6MWT, TUG (S)
Tenconi 2021 [94] (2)	X	X	X		X	X	X		Prehab outcomes (T0 to T1) not reported; **++ 48.8 m 6MWT** baseline to 6 months; −3.05 m at 1 month post‐op compared to −29.41 m for controls (P)
*Post‐op*									
Liu 2022 [137] (3)		X			X				NS 6MWT (S)
Jonsson 2019a, 2019b [101, 124] (2)		X			X		X		NS 6MWT (P)
Brocki 2016 [98] (2), 2018 [97] (2)		X					X		NS 6MWT (2016) & EQ‐5D‐5L Mobility Scale (2018) (S)
Li 2021 [101] (2)		X					X	Pulmonary exercise, animation, education	+ 17.2 m 6MWT (S)
Yang 2018 [103] (3)		X					X	Self‐efficacy	**+ 100.2 m 6MWT** (both groups declined, exercise better than control)
Cheng 2022 [99] (3)		X			X	X	X		**+ 58 m 6MWT** (P)
Arbane 2014 [96] (2)		X	X		X	X			NS incremental shuttle walk (S)
*During or immediate post‐treatment*									
Stigt 2013 [119] (2)			X		X	X			**++ 94 m 6MWT** at 3 months (S)
Tatemansu 2021 [120], Naito 2019 [112] (3)			X			X		Nutritional counseling	NS 6MWT or 5 m gait speed; ++ 5XSTS (*p* < 0.05) (8 weeks) (P)
Ahn 2021 [104] (3)			X		X	X			+ 27 m 6MWT (pre post) (P), feasibility (S)
Quist 2018 [116] (2)			X		X	X		Health behavior counseling	+, ++ 25–28 m within group 6MWT, NS difference early vs. late rehab groups (S)
Edvardsen 2015 [108] (2)			X		X	X			+ 2.1 Chair stand and 4.3 stair run (S)
Edbrooke 2019 [107] (2)			X		X	X		Behavior change strategies	NS 6MWT short (9 weeks) and long‐term (6 months) (P)
Ester 2021 [109] (3)			X		X	X		Education, nutrition	NS 6MWT (S) feasibility
Quist 2020 [115] (2)			X		X	X		Relaxation	NS 6MWT (S) + 41 m 12 weeks
Quist 2015 [121] (3)			X		X	X		Relaxation	**+ 33.6 m 6MWT** (pre post) (S)
Kendall 2020 [110] (3)			X				IMT vs. IMT + EMT vs. EMT		NS 6MWT
Andersen 2013 [105] (3)			X		X		X	—	NS incremental shuttle walk (P)
Liu 2021 [102] (2)			X		X	X	X		**+ 80 m**, **++ 42 m** 6MWT (P)
Ozalevli 2010 [113] (4)			X		X	X	X		**+ 75 m 6MWT** (pre post) (S)
Rutkowska 2019 [117] (2)			X		X	X	X	Relaxation	**+ 41 m 6MWT**, **+** up and go, +1 repetition 30 s chair stand (P)
Dogan 2020 [106] (2)			X					Acupressure	**+ 60 m 6MWT** (S)
Milbury 2019 [111] (2)			X					Yoga dyads	++ d = 0.94 6MWT (P) + d = 1.07 6MWT
Salhi 2015 [118] (2)			X		X	X		Whole‐body vibration	**+ 38 m 6MWT** aerobic & strength, NS adding vibration
Park 2019 [114] (2)			X		X	X		Mobility app	**+ ++ 68.0 m 6MWT**
*Survivorship*									
McDonnell 2020 [135] (3)				X			X	Yoga, meditation	NS Qualitative improvement in symptoms and 6MWT in higher level of yoga program

*Note:* + = short‐term gain; ++ = long‐term gain; > = 4 weeks post intervention; bolded = clinically meaningful finding. Outcomes: 6MWT = six‐minute walk test; 5X STS = five times sit‐to‐stand test; EQ‐5D‐5L = 5‐Level EuroQol Five Dimension Questionnaire.

Abbreviations: EMT = expiratory muscle training; IMT = inspiratory muscle training; m = meters; NS = non‐significant; P = primary; S = secondary.

### 2.7 Recommendations

The Grading of Recommendations Assessment, Development and Evaluation (GRADE) scale was employed for evaluating the combined evidence being synthesized [78, 79, 80]. Evidence was aggregated on a 4‐point scale as high (A), moderate (B), low (C), and very low (D) respectively. Conflicting evaluations were discussed and resolved among all authors. Harmonization of ratings was achieved by group review, and consensus was reached on all ratings and classifications. Recommendations were generated for evidence found at A–C grades; D grades were not considered in making recommendations. Once the reviews were complete and recommendations compiled, the manuscript was reviewed by peers, including a medical oncologist, an individual living with or beyond cancer, a cancer rehabilitation physiatrist, and an exercise physiologist, whose input informed completion of this work.

## 3 Results

**FIGURE 1:**
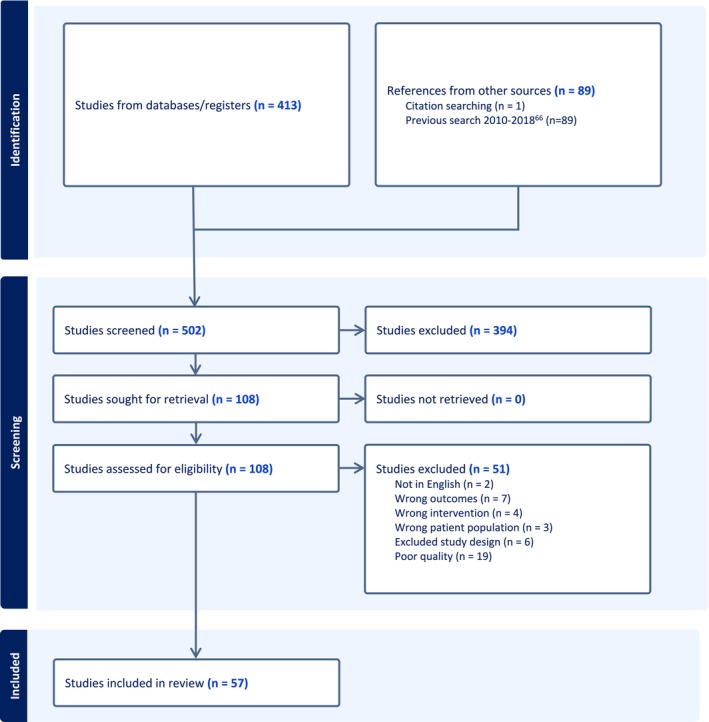
PRISMA flow diagram.

Fifty‐seven articles are included in the final sample representing 54 trials (Figure 1). Of the final included studies, 40 were controlled trials (42 manuscripts), 11 were pre/post studies (12 manuscripts), 2 were cohort studies, and 1 was a case series. The studies encompass a total of 4612 subjects (3933 analyzed), with 2630 subjects (2217 analyzed) having received an intervention. Forty of the trials focused on surgically treated patients, 13 evaluated patients with advanced disease and/or inoperable status, and 1 examined long term survivors; however, at least 10 of these studies included a full range of disease stages. Full data extraction can be found in Supplemental Content—Appendices [Link], [Link], [Link]. Data are summarized in Table 5, and final recommendations are compiled in Table 6. Quality reviews for the studies are found in Supplemental Content—Appendices [Link], [Link], [Link], [Link]. Most studies focus on non‐small cell lung cancer (NSCLC), though some samples contain small numbers of patients with small cell lung cancer, or unspecified lung cancer type.

### 3.1 State of the Literature

#### 3.1.1 Prehabilitation Phase

Prehabilitation and mobility outcomes were assessed in 16 studies including an aerobic exercise intervention, most incorporating strengthening (9) and/or breathing (11) exercises. This literature focuses mostly on presurgical patients, that is, those with Stage I–III disease. Eleven articles (ten trials) examined the prehabilitation phase exclusively [29, 81, 82, 83, 84, 85, 86, 87, 88, 89, 90], whereas other trials incorporate prehabilitation in combination with later phase interventions [91, 92, 93, 94, 95, 96]. In several studies, the prehabilitation component was minor compared to the later interventions [92, 94, 96]. Some programs featured a short duration of 7 days [83, 84, 85, 93], whereas most studies consisted of 2‐ to 4‐week interventions. A majority of studies indicated intensity parameters in the moderate to vigorous range, though exact methodology varied.

#### 3.1.2 During and Immediate Post‐Treatment Phase

Acute surgical post‐operative interventions for improving mobility were studied exclusively in 7 trials reported in 9 papers [97, 98, 99, 100, 101, 102, 103, 104, 138] while studies that provided intervention across multiple time periods, but included surgical post‐operative care as one component, was investigated in five studies [92, 93, 95, 96, 97]. Eighteen studies (19 papers) investigated interventions exclusively provided in the During and Immediate Post Treatment phase (such as radiation or chemotherapy) [103, 105, 106, 107, 108, 109, 110, 111, 112, 113, 114, 115, 116, 117, 118, 119, 120, 121, 122], while four studies [91, 92, 94, 95] investigated prehabilitation and intervention during or immediate post treatment. Nine of these investigated a combined aerobic and strength training program, one investigated aerobic and breathing exercise, one investigated breathing exercise only, while three trials combined all three modalities of intervention. No studies investigated aerobic exercise in isolation. Physical activity, such as step counts, was examined in eleven studies (13 papers) primarily in the During and Immediate post‐treatment period [82, 91, 97, 99, 101, 108, 110, 111, 113, 121, 123, 124, 125]. Of these, most included a combination of aerobic and resistive interventions. Thirty‐two studies examined general function [83, 84, 85, 87, 89, 92, 96, 97, 105, 106, 109, 111, 112, 115, 116, 117, 118, 119, 120, 122, 123, 125, 126, 127, 128, 129, 130, 131, 132, 133, 134, 135] and sixteen [91, 106, 110, 112, 115, 116, 117, 122, 123, 125, 126, 127, 128, 131, 133, 134] assessed social function.

Nineteen studies (18 trials) included patients with Stage IIIC or IV disease, either comprising the entirety of the sample [98, 99, 110, 113, 114, 116, 118, 121, 122, 123, 125, 126, 127] or as part of mixed populations [106, 107, 108, 115, 129]. Literature regarding rehabilitation interventions in the survivorship phase was extremely sparse, with only one study [135] including individuals in this period. In general, a lack of consistent harms was found. Complications were few and are listed in Appendices [Link], [Link], [Link]. Arbane et al., in an early postoperative hospital‐based exercise study (subjects exercising to 60%–90% of heart rate reserve and performing 10 repetition maximum weight lifting, to Borg rating of perceived exertion of 13–15 [96]) reported increased cardiac events (8% of intervention subjects versus 0% of controls), but no differences in survival (1 death in each group), nor differences in total complication rates between intervention and control groups at 33% vs. 31%, respectively, with the intervention group having a lesser frequency of respiratory complications (16% vs. 24%); these observations were not further evaluated descriptively or statistically. Edvardsen et al. [108] noted an occurrence of a hip fracture during balance training. Several other studies note presence of joint or muscle pains [83, 113, 121], plantar aponeurositis [113, 121], or dyspnea on exertion [113, 121]. Conversely multiple studies note safety benefits including fewer pulmonary or other medical complications [85, 100], and fewer emergency department visits [114].

### 3.2 Mobility (Table 1)

A. Prehabilitation Phase

*Recommendations for Mobility in the Prehabilitation Phase:*

- Combined aerobic and other exercise (strengthening and/or breathing) should be used to improve mobility in the prehabilitation phase (Grade B).
- Breathing exercise should not be employed as the only type of exercise intervention to improve mobility in the prehabilitation phase (Grade B).

#### 3.2.1 Evidence Summary

##### 3.2.1.1 Combined Intervention Studies (Grade B)

Most prehabilitation studies examining mobility outcomes employed multimodal exercise interventions, including aerobic + strengthening exercise [81, 86, 89, 91, 92], aerobic + breathing exercise [84, 85, 90, 96], and aerobic + strengthening + breathing exercise [83, 87, 95]. The majority of these studies showed significantly improved outcomes at the end of the prehabilitation phase, including six good quality Level 2 studies among seven papers [81, 83, 85, 86, 87, 91, 137], three fair quality Level 2 studies [84, 89, 93], and one good quality Level 3 study [89]. Of the nine studies showing significantly favorable outcomes, five of them reached the clinically meaningful range of a six‐minute walk test (6MWT) outcome measure, including one good quality Level 2 study for aerobic + strengthening exercise [85], one good quality Level 2 study [84] and one fair quality Level 2 study [89] for aerobic + breathing exercise, and two good quality Level 2 studies for aerobic + strengthening + breathing exercise [83, 87]. Some fair quality studies included a prehabilitation phase and a later intervention phase but did not separately report prehabilitation outcomes and are thus indeterminate for prehabilitation and mobility outcomes [92, 94, 95, 96].

##### 3.2.1.2 Breathing Exercise (Grade B)

Notably, two studies included intervention arms independently examining effects of breathing exercise, including two Level 2 studies [83, 85]. These two studies showed benefit for mobility in their combined aerobic and breathing exercise arms, but mobility benefits did not reach significance in their breathing exercise‐alone arms. Based on these findings, breathing exercise as an isolated approach cannot be recommended as adequate to improve mobility in prehabilitation programs.

##### 3.2.1.3 Aerobic Exercise Alone (Grade D)

Aerobic exercise was a prominent component of most prehabilitation studies, with aerobic exercise approaches including walking [90, 92, 95], treadmill [85, 89, 93], NuStep [83, 84, 85], cycling [85], and mixed options or unspecified [82, 88, 91, 94, 96]. Aerobic exercise received limited attention as an isolated prehabilitation intervention, confined to a single study which had non‐significant findings [81]. Overall, despite aerobic exercise being a virtually ubiquitous component of lung cancer prehabilitation studies, it has been insufficiently studied as an isolated intervention to allow a recommendation to be generated.

##### 3.2.1.4 Other Interventions (Grade D)

Five studies examined other interventions within the prehabilitation phase, including three level 2 studies [87, 89, 91] and one Level 3 study [87], with two of the studies [87, 88] demonstrating significant benefit. In all the studies, the interventions were adjunctive to multimodal exercise interventions and included diet and nutrition strategies, as well as coping and relaxation skills. Given the variety of “other interventions” and the adjunctive nature of how they are incorporated into study design, no conclusions can be made specific to the prehabilitation phase with regard to mobility, for measures including psychological and stress management, and diet and nutrition strategies.

##### 3.2.1.5 General Observations of the Prehabilitation Literature

Most prehabilitation studies did not examine post‐treatment (typically postsurgical) time points in their mobility outcomes, but two good quality Level 2 studies that did found significant benefit, including at day of hospital discharge [84] and 30 days postoperatively [86]. Many of the studies in this group examined non‐mobility postsurgical parameters as their primary outcome measure, such as complication rates and/or hospital length of stay, often with favorable findings, though these outcome measures are outside of the defined scope of the current guideline. A majority of the studies that demonstrated clinically meaningful benefits for prehabilitation mobility outcomes specified supervised intervention [83, 85, 86].

B. Surgical Post‐Operative Acute Phase

*Recommendations for Mobility in the Surgical Post‐Operative Acute Phase:*

- Available evidence lacks consensus to make a recommendation for a combined exercise intervention to improve mobility during the surgical post‐operative acute phase.

#### 3.2.2 Evidence Summary

##### 3.2.2.1 Combined Exercise (Grade B)

Nine articles describing seven studies investigated the impact of short‐term, in‐hospital interventions for improving mobility after lung cancer surgery (six Level 2 [97, 98, 99, 101, 102, 124]; three Level 3 [100, 104, 138]). Aerobic, strength, and breathing exercises were investigated in different combinations; clinically meaningful change occurred in two trials that incorporated breathing exercise alone or with aerobic and resistive training [103, 104]. However, not all trials that incorporated breathing exercises resulted in statistically significant and/or clinically meaningful findings.

C. During and Immediate Post‐Treatment Phase

*Recommendations for Mobility in the During and Immediate Post‐Treatment Phase:*

- A combined program of aerobic, resistance, and breathing exercise should be used to improve mobility in the During and Immediate Post‐treatment Phase. (Grade B)

#### 3.2.3 Evidence Summary

##### 3.2.3.1 Combined Exercise (Grade B)

Seventeen articles (16 studies [10 Level 2 [97, 103, 108, 109, 115, 116, 117, 118, 119, 120], 6 Level 3 [105, 106, 110, 113, 121, 122], and 1 Level 4 [113]]) investigated the impact of aerobic, strength, and/or breathing exercise on patients being treated for lung cancer with surgical, radiation, or chemotherapy treatment. Combined aerobic and strength training yielded significant positive differences on the 6MWT in 7 of 10 studies; of those only 3 demonstrated clinically meaningful change [119, 120, 122]. However, all three of the trials (2 Level 2 [103, 118], 1 Level 4 [113]), investigating the impact of a combined aerobic, resistance, and pulmonary exercise intervention in operative [102] and non‐operative populations [114, 118] found both statistically and clinically significant differences in 6MWT distances.

#### 3.2.4 Survivorship Phase

A lone single study examined the effect of breathing and yoga/meditation on mobility in the survivorship phase and found no significant changes in the 6MWT [135].

### 3.3 Physical Activity (Table 2)

**TABLE 2:** Physical activity.

Author, year (level)	Prehab	Surgical post‐op acute	During or immediate post‐treatment	Survivor‐ship	Aerobic	Strength	Breathing	Other intervention	Physical activity impact (see key below)
Finley 2021 [81] (3)	X				X				NS change in moderate/vigorous activity
Ferreira 2021 [90] (2)	X		X		X	X			NS self‐reported energy expend/week on CHAMPS
Brocki 2018 [97] (2)		X							+ 16% PAS improved sedentary activity (P)
Jonsson 2019a, 2019b [101, 124] (2)		X			X		X	Range of motion exercise	+ 14 steps/h accelerometer, short‐term (P) NS longer‐term accelerometer and steps/h; IPAQ‐E (P)
Arbane 2014 [96] (2)		X	X		X	X			NS Accelerometer 4 weeks post‐surgery (P)
Bade 2021 [122] (2)			X		X			Education, relaxation, texting	**+ Self‐reported activity 88 min/week (S)** NS change in step counts in Int group only (S)
Tatemansu 2021 [120]; Naito 2019 [112] (3)			X			X		Nutritional counseling	++ time in moderate‐vigorous activity (increase of 2.3min/day), + daily step counts (571) (P)
Edbrooke 2019 [107] (2)			X		X	X		Behavior change strategies	NS Self‐report IPAQ and accelerometry (S)
Ester 2021 [109] (3)			X		X	X		Education, nutrition	NS pre‐post Godin (S) (feasibility)
Kendall 2020 [110] (3)			X				IMT vs. IMT + EMT vs. EMT	General exercise	+ Accelerometry (65.3 IMT vs. 31.1 EMT over control) counts/min and sedentary with IMT>EMT (S)
Maddocks 2013 [124] (2)			X					Neuromuscular electrical stimulation	NS accelerometry (S)

*Note:* + = short‐term gain; ++ = long‐term gain; > = 4 weeks post intervention; bolded = clinically meaningful finding. Outcomes: CHAMPS = Community Healthy Activities Model Program for Seniors (questionnaire); PAS=Physical Activity Scale; IPAQ = International Physical Activity Questionnaire; IPAQ‐E = Modified International Physical Activity Questionnaire for the Elderly.

Abbreviations: EMT = expiratory muscle training; IMT = inspiratory muscle training; NS = non‐significant; P = primary; S = secondary.

*Recommendations for Physical Activity in the Prehabilitation Phase:*

- Insufficient evidence is available to make a recommendation for interventions to improve physical activity during the Prehabilitation Phase.

A. Prehabilitation Phase

#### 3.3.1 Evidence Summary

##### 3.3.1.1 Combined Intervention Studies (Grade D)

One small level 3 fair quality study [91] assessed unsupervised home‐based aerobic and strengthening exercise in 18 lung cancer patients before and during treatment, employing a pedometer, with the intervention of 5 video conferenced physical and occupational therapy sessions, one of which was at least 7 days preoperative. Median step count was 5145 preoperatively, 1331 during hospitalization, and 2570 at 2–4 weeks postoperatively. Limitations of this study included that it was a feasibility study, without a control group, and without pre‐ and post‐activity outcome measurement within the prehabilitation phase specifically. Additionally, only a minor component of the intervention was within the prehabilitation phase. Ferreira et al. [90] found no significant differences between prehabilitation plus rehabilitation and rehabilitation groups in self‐reported energy expenditure per week in a home‐based program, though near‐significance for prehab + rehab (112.6 vs. 74 kcal/kg/week; *p* = 0.07) at the pre‐operation time point was seen.

##### 3.3.1.2 Aerobic Exercise Alone (Grade D)

Finley et al. [81] examined adherence to a home‐based moderate to vigorous 4‐week prehabilitation exercise program, utilizing an exercise prescription and provision of an activity tracker, finding 18 of 30 subjects having data associated with their tracking device. On only 16% of days was the target of 30 min moderate to vigorous activity achieved and an average of 20 min of moderate‐vigorous activity was seen. Once an outlier was removed, the average dropped to 10 min. Overall, this home‐based Level 3 study showed modest effects of unclear significance.

*Recommendations for Physical Activity in the Surgical Post‐Operative Acute Phase:*

- Combined intervention may be used to improve physical activity in the Surgical Post‐operative Acute Phase. (Grade C)

B. Surgical Post‐Operative Acute Phase

#### 3.3.2 Evidence Summary

##### 3.3.2.1 Combined Interventions (Grade C)

Two studies (3 papers) reported short‐term outcomes (4 weeks post‐operatively [96] vs. 5 days post‐operatively [101, 124]) following interventions that combined aerobic exercise (bicycle or walking), strength training [96], shoulder girdle and torso range of motion [123], and breathing exercise [123]. The study by Arbane et al. [96] also included continuation of the intervention past hospitalization phase. While physical activity was not significantly improved by aerobic plus resistance training in the study by Arbane et al. [96], Jonsson et al. [123] reported an increase in (average) 495 steps per day in the intervention group with a combined aerobic, breathing, and range of motion program. The improvement in step counts by Jonsson et al. [123] was short of the low confidence level for clinically meaningful difference of 600 steps per day.

##### 3.3.2.2 Breathing Exercise (Grade D)

Brocki et al. [97] investigated the impact of a twice daily inspiratory training intervention during hospitalization for lung surgery, with a follow‐up phone coaching session after discharge. The intervention, which was compared to a usual care control group, did not produce significant differences in physical activity at 2 weeks.

*Recommendations for Physical Activity in the During and Immediate Post Treatment phase:*

- A multimodal program including exercise and education may be used to improve short‐term physical activity levels in individuals with late‐stage cancer. (Grade B)

C. During and Immediate Post‐Treatment Phase

#### 3.3.3 Evidence Summary

##### 3.3.3.1 Combined Interventions (Grade B)

Two studies investigated the impact of combined aerobic and strength training [97, 108] during or shortly after cancer intervention. Neither of these relatively low intensity interventions made a significant improvement in physical activity outcomes. Kendall et al. [110] did find a significant improvement in physical activity when inspiratory muscle training was added to their usual care protocol of general exercise with aerobic and resistance training components. All three of these trials were conducted in populations of mixed staging.

Impacting physical activity levels in advanced cancers was the focus of four trials (five articles) [110, 113, 121, 123, 125]. One study [122] demonstrated a clinically significant increase in weekly physical activity (+88 min/week intervention vs. control) (*p* = 0.05) and an increase in 898 steps/day at 6 weeks in the intervention group (aerobic exercise, relaxation training, and education) as compared to control. However, activity dropped to baseline levels by 12 weeks. Ester et al. [109] used a multimodal intervention (aerobic, strength training, flexibility, education, and palliative care) but no significant improvement in activity levels was demonstrated. Other interventions such as neuromuscular stimulation and breathing training have been investigated yet have not demonstrated clinically significant impact on physical activity.

### 3.4 General Function (Table 3)

A. Prehabilitation Phase

*Recommendations for General Function in the Prehabilitation Phase:*

- Insufficient evidence is available to make a recommendation for interventions to improve general function during the Prehabilitation Phase. (Grade C–D)

#### 3.4.1 Evidence Summary

Among the eleven studies examining prehabilitation and general function [83, 84, 85, 87, 89, 91, 92, 95, 96, 126, 128] there were a paucity of significant findings, however most of the studies had general function as a secondary outcome and/or consisted of short duration interventions of 1–2 weeks. Also, nearly half of the studies were not limited to the prehabilitation phase and often emphasized later phases in their design [91, 96, 126, 128, 129].

##### 3.4.1.1 Breathing Exercise (Grade C)

Four prehabilitation‐only studies measuring general function included breathing exercise intervention [83, 84, 85, 87]. All were mixed‐intervention studies, but one good quality Level 2 supervised study included a breathing‐exercise only arm (compared to mixed intervention and to non‐exercising controls) [82]. At least two studies explicitly focused on high‐risk patients [85, 126]. Three of the four studies were of short duration (7 days) [83, 84, 85], and the other consisted of a two‐week intervention [86]. None of these studies demonstrated benefit of the intervention, including the study examining breathing exercise as an isolated intervention.

##### 3.4.1.2 Balance Exercise (Grade D)

Only one prehabilitation study measuring general function included balance as part of the intervention, which also included aerobic, strengthening, and flexibility exercises [128]. This was a gero‐oncologic study enrolling mostly Stage IV patients awaiting and starting chemotherapy. This study was conducted in both outpatient and home settings, with mixed supervision. Significant findings with regard to general function were not seen in this 10‐week study, though the authors pointed to potential utility of the Fried Frailty scale in detecting change. No studies examined balance exercise as the sole intervention.

##### 3.4.1.3 Other Interventions (Grade D)

Six prehabilitation studies measuring general function included other rehabilitation interventions. These interventions included web‐based education [125], mind–body‐spirit therapy with cognitive behavioral therapy as control [127], nutritional supplementation [87, 91], flexibility [89, 129] and stress or anxiety management [89, 91]. Outcome measures in this group included European Organisation for Research and Treatment of Cancer (EORTC) scales [89, 91, 126, 128], the 36‐item short form survey (SF‐36) [89, 91], the World Health Organization Disability Assessment Schedule (WHODAS) [86], the Functional Assessment of Cancer Therapy‐General (FACT‐G) [127], Fried Frailty Scale [128], the Geriatric‐8 (G8) Health Status Assessment Tool [128], and Vulnerable Elders Survey‐13 (VES‐13) [128]. Most of the studies used the interventions adjunctively, with exercise as the main intervention [87, 89, 91, 129], but two of the studies had the “other” invention as the primary intervention [126, 128].

Findings reached significance in one fair quality Level 2 study [127], which consisted of a supervised mind body spirit intervention, though only about 10% of subjects in this study were in the prehabilitation phase (15/157 total subjects and 8/76 min/body/spirit subjects), and results were not presented according to phase of treatment. Therefore, actual impact of the intervention within the prehabilitation phase was unclear in this study. Additionally, there was not a true non‐intervention control group, with the control subjects having received cognitive‐behavioral therapy. Both groups had improved function per the EORTC, but a larger effect was seen in the mind body spirit group. Huang et al. [125] (level 2) reported on a home‐based study of web‐based education and text messaging as the primary intervention, and which did not show significant findings [125].

##### 3.4.1.4 Combined Intervention Studies (Grade D)

Most but not all available evidence suggests that combined intervention programs in the prehabilitation phase may not be beneficial for general function outcomes. Seven studies examined general function outcomes of prehabilitation combined interventions, all with general function as a secondary outcome. A home‐based study by Ferreira et al. [90] comparing prehab + rehab to rehab‐only groups found significantly favorable findings in their prehabilitation + rehabilitation group, compared to rehabilitation alone, for SF‐36 Total at 4 weeks postoperatively, and Physical Summary scores at 4 and 8 weeks postoperatively. On the other hand, other prehabilitation studies have not shown favorable general function effects. Two good quality Level 2 studies [83, 87] examined the combination of aerobic, strengthening, and breathing exercise, and one of them [86] also included nutritional (whey supplementation) and psychological (relaxation) components, but neither yielded significant findings. Another good quality Level 2 study [84] examined aerobic and breathing exercise as did a fair quality Level 2 study [81] without significant findings. One fair quality Level 2 study [88] examined aerobic and strengthening exercise, combined with stress management and nutritional approaches and also failed to find significant improvements in general function outcomes. Outcome measures varied across studies and included SF‐36 [88], EORTC Physical Function [83, 85], WHODAS [86], or VES‐13 [128]. The length of the prehabilitation intervention may be a factor impacting outcomes. Three Level 2 studies [83, 84, 85] investigated a 1‐week intervention, and another [86] used a 2‐week intervention with none reaching significance. Whereas the Ferreira et al. [90] study found significant improvement in general function with a longer prehabilitation intervention combined with post‐operative exercise (4 weeks prehabilitation and 8 weeks postoperative exercise). The level of exercise supervision varied as well. The majority of studies consisted of supervised interventions [83, 84, 85] however others were home‐based interventions [87, 91] or involved mixed [128] or uncertain [88] supervision. One study [86] showing significant benefit for the mobility outcome measure incorporated a physical activity diary in its methodology but did not provide analysis of that information.

*Recommendations for General Function in the Surgical Post‐Operative Acute Phase:*

- Insufficient evidence is available for intervention effects on General Function in the Surgical Post‐Operative Acute Phase.

B. Surgical Post‐Operative Acute Phase

#### 3.4.2 Evidence Summary

Five studies investigated interventions occurring in the acute post‐operative period, although all included additional intervention in either the prehabilitation or during/immediate medical treatment phases [92, 95, 128, 129]. Thus, no evidence on the impact of postoperative acute treatment alone on general function was found.

*Recommendations for General Function in the During and Immediate Post‐Treatment Phase:*

- Combined exercise programs including aerobic, strength, and breathing training combined with other supportive care interventions may be used to improve general function in the During and Immediate Post‐Treatment Phase. (Grade B)
- Aerobic training alone may not be sufficient to improve self‐reported general function in the During and Immediate Post‐Treatment Phase. (Grade C)

C. During and Immediate Post‐Treatment Phase

#### 3.4.3 Evidence Summary

Nineteen studies [105, 106, 109, 111, 112, 115, 116, 117, 119, 120, 122, 123, 125, 127, 130, 131, 133, 134, 135] investigated the impact of different interventions given during or immediately after medical treatment on general function outcomes, nine of which were rated as Level 2 (Table 3).

**TABLE 3:** General function.

Author, year (level)	Prehab	Surgical post‐op acute	During or immediate post‐treatment	Survivor‐ship	Aerobic	Strength	Breathing	Other intervention	General function impact (see key below)
Lai, Su, Qui 2017 [84] (2)	X				X		X		NS EORTC‐QLQ‐C30 physical function (S)
Lai, Huang, Yang 2017 [83] (2)	X				X		X		NS EORTC‐QLQ‐C30 physical function (S)
Morano 2014 [88] (2)	X				X	X		Stress management, nutrition, stretching	NS SF‐36 physical; + 0.67 UULEX (S)
Huang 2017 [125] (2)	X				X	X	X		NS EORTC‐QLQ‐C30 physical function (S)
Liu 2020 [86] (2)	X				X	X	X	Whey supplement; relaxation	NS WHODAS 2.0 (S)
Van der Leeden 2019 [95] (3)	X	X			X		X		**+ 10.0 SF‐36 physical function** between 6 weeks and 3 months; not back to baseline at 3 months; no control group (S)
Ferreira 2021 [90] (2)	X		X		X	X		Education, nutrition, address anxiety	At 4 weeks, + 8.5 SF‐36 Physical Summary score (*p* = 0.006) and + 7.2 SF‐36 total (*p* = 0.022). At 8 weeks, + 7.4 for Physical Summary score (*p* = 0.034). Physical Function subscale NS between prehab + rehab (71) and rehab‐only (59.3), *p* = 0.065 at 4 weeks
Huang 2019 [125] (2)	X		X					Web‐based education	NS EORTC‐QLQ‐C30 physical (P)
Martinez‐Velilla 2021 [128] (3)	?	Uncertain	X		X	X		Balance and flexibility exercise	NS VES (S)
Lafaro 2020 [91] (3)	X	X	X		X	X			**+ 1.5 SPPB** (S)
Lau 2020 [127] (2)	X	X	X	Uncertain				Mind, Body, Spirit (includes some physical exercise) vs. Cognitive Behavioral Therapy	**+**, **++**; **4.4 vs. 4.1 FACT‐G function** (CM) and **11.9 vs. 9.0 EORTC‐QLQ‐C30 physical** 1 week post. Both groups improved, larger effect size in mind body spirit group and better maintained (P/S unspecified)
Tenconi 2021 [94] (2)	X	X	X		X	X	X		NS SF‐12 physical function
Arbane 2014 [96] (2)		X	X		X	X			NS SF‐36 physical function; **+ prevented decline** in air flow obstruction subgroup (S)
Hwang 2012 [126] (2)			X		X				NS EORTC‐QLQ‐C30 physical
Bade 2021 [122] (2)			X		X			Education, relaxation, texting	NS EORTC‐QLQ‐C30 physical function (S)
Ahn 2021 [104] (3)			X		X	X			NS EORTC‐QLQ‐C30 (S)
Stigt 2013 [119] (2)			X		X	X			NS SF‐36 general or physical
Park 2019 [114] (2)			X		X	X		Mobility app	++ 2.9 EORTC‐QLQ‐C30 physical function
Quist 2015 [121] (3)			X		X	X		Relaxation	NS pre‐post FACT‐G physical and functional (S)
Quist 2018 [116] (2)			X		X	X		Health behavior counseling	NS EORTC‐QLQ‐C30 physical function (S)
Quist 2020 [115] (2)			X		X	X		Relaxation	NS FACT‐G physical function (S)
Sommer 2020 [133] (2)			X		X	X		Nutrition, smoking cessation, health behavior counseling	+ (1.6 early, **2.0** late), ++ (1.7 early, 0.8 late) **FACT‐G functional** with early or late intervention; NS between groups
Salhi 2015 [118] (2)			X		X	X		Whole‐body vibration	NS EORTC‐QLQ‐C30 physical (S)
Sui 2020 [134] (2)			X		X	X		App‐based education, balance, psychological support	**+ 3.9 between‐group; 11.97 within‐group increase for intervention EORTC‐QLQ‐C30 function**
Kendall 2020 [110] (3)			X				IMT vs. IMT + EMT vs. EMT		Improved in both IMT and IMT + EMT groups (*p* = 0.003, 0.008) Euro‐QOL usual function
Andersen 2013 [105] (3)			X		X		X		NS EORTC‐QLQ‐C30 physical function (S)
Messaggi‐Sartor 2019 [129] (3)			X		X		X		NS EORTC‐QLQ‐C30 physical (S)
Edvardsen 2015 [108] (2)			X			X	X		+ 8.5 SF‐36 physical (between group difference), NS 1 leg balance (S)
Milbury 2019 [111] (2)			X					Yoga	**+ 8.1 SF‐36 general function** (between group difference) (S)
Raz 2016 [130] (4)			X					Supportive care sessions, psychological, social, spiritual	**++ 2.6 FACT‐G functional** (P)
Schofield 2013 [132] (3)			X					Tailored supportive care, education	NS EORTC‐QLQ‐C30 physical
Maddocks 2013 [124] (2)			X					Neuromuscular electrical stimulation	NS EORTC‐QLQ‐C30 physical function (S)

*Note:* + = short‐term gain; ++ = long‐term gain; > = 4 weeks post intervention; bolded = clinically meaningful finding. Outcomes: EORTC‐QLQ‐C30 Q‐30 = European Organisation for Research and Treatment of Cancer‐Quality of Life Questionnaire‐Core 30; FACT‐G = Functional Assessment of Cancer Therapy‐General; SF‐36 = Short Form 36 Item Questionnaire; UULEX = Unsupported Upper Limb Exercise Test; WHODAS 2.0 = World Health Organization Disability Assessment Scale; SPPB = Short Physical Performance Battery; VES = Vulnerable Elders Scale.

Abbreviations: NS = non‐significant; P = primary; S = secondary.

##### 3.4.3.1 Combined Training (Grade B)

Twelve studies (eight Level 2 [109, 115, 116, 117, 119, 120, 134, 135], and four Level 3 [105, 106, 122, 130]), investigated the impact of combined interventions that included aerobic, resistance, breathing, and/or other supportive care services on functional outcomes. Ten of the studies combined aerobic and resistance training [105, 115, 116, 117, 119, 120, 122, 127, 134, 135], two investigated aerobic and breathing exercise [106, 130], and one combined resistance and breathing exercise [108]. Four of the eight Level 2 studies demonstrated statistically significant improvements in functional outcomes [109, 115, 134, 135] of which two were clinically important differences [134, 135].

Sui et al. [134] and Sommer et al. [133] demonstrated clinically significant impact on self‐reported physical function of combined aerobic and resistance training with other supportive care interventions. The program investigated by Sui et al. [134] was based on an app used for a year‐long period, and Sommer et al. [133] included nutritional and health behavior counseling with aerobic exercise. Edvardsen et al. [108] found that combined high‐intensity aerobic, resistance, and breathing training also could improve self‐reported physical function, when completed three times per week for 20 weeks. The remainder of studies failed to find a significant difference in general function outcomes with combined aerobic and resistance training [105, 116, 117, 120, 122]. For example, in Quist et al.'s [121] combined exercise intervention delivered over 6 weeks, no significant difference in self‐reported physical function was found, potentially indicating that longer term interventions are needed to improve general function outcomes [121]. However, this study may have been underpowered to find such differences. Similarly, Stigt et al. [119] did not find a treatment effect in their surgically treated population, but this study did not meet target enrollment, having been closed early due to the introduction of video‐assisted surgery.

##### 3.4.3.2 Aerobic Training (Grade C)

Two studies investigated the impact of aerobic training on function, although this was a secondary outcome for both studies [123, 127]. One pilot study [122] investigated the primary impact of walking with education, relaxation training, and text supports. Self‐reported function was not found to differ from a usual care control group. When high‐intensity aerobic training was compared to usual care (including some who received resistance exercises) in a separate study [126], there were no significant differences between groups on self‐reported function.

##### 3.4.3.3 Other Interventions (Grade C)

Four studies investigated the impact of other interventions including yoga [111], supportive care [131, 133], and neuromuscular stimulation [124]. Millbury et al. [111] investigated the impact of a yoga intervention delivered two to three times per week for 6 weeks to both patients and a family caregiver. Patients with either lung or esophageal cancer who received this intervention reported a clinically important and statistically significant improvement in physical function as compared to waitlist controls. Other supportive care interventions had mixed results, with one Level 4 study reporting a program of sessions focusing on psychological, social, and spiritual needs demonstrating improvements in functional reports [130].

##### 3.4.3.4 Mixed Prehabilitation and During and Immediate Post‐Treatment Intervention (Grade C)

Six studies (four Level 2 [91, 95, 126, 128] and two Level 3) [92, 129] investigated the impact of interventions initiated pre‐medical treatment and continued during cancer treatments. Lafaro et al. [91] investigated the impact of a physical and occupational therapy telerehabilitation intervention initiated pre‐operatively and continued up to 2 weeks post‐discharge. While designed as a feasibility study, short‐term improvements in the short physical performance battery (SPPB) and timed up and go (TUG) were shown. Martinez‐Velilla et al. [128] investigated the impact of a combined aerobic, resistance, balance, and flexibility exercise program on participants with newly diagnosed NSCLC. The intervention was initiated twice weekly and continued during treatment for 10 weeks. Based on the Vulnerable Elders Scale (VES), no difference was seen between those receiving intervention and a control group receiving usual care. Huang et al. [125] investigated the impact of a web‐based health education program including symptom management and supportive care initiated prior to planned chemotherapy and continued during treatment. No change in physical, cognitive, social, or role function outcomes were found when compared to a usual care control group, though improvements in global QOL and emotional function were seen. Another mixed‐phase study, Ferreira et al. [90], is discussed above under prehabilitation and general function, as its design differentiates effects of prehabilitation versus rehabilitation‐only interventions. While the rehabilitation intervention demonstrated a clinically meaningful change, the addition of a prehabilitation component did not significantly improve outcomes. Tenconi et al. [94] investigated a combined exercise intervention that started in the prehabilitation phase and continued 8 weeks after surgery. While walking distance did improve, their secondary outcome of self‐reported physical function did not change significantly. Lastly, Lau et al. [127] demonstrated that a mind, body, spirit intervention which included some physical exercise improved self‐reported physical function greater than a cognitive behavioral therapy intervention.

##### 3.4.3.5 Interventions Completed in Groups at Different Time Points (Grade C)

Lau et al. [127] investigated the impact of a dyadic body–mind–spirit (BMS) intervention as compared to a cognitive behavioral therapy (CBT) intervention with participants who were in any stage of treatment, including pre‐treatment, during treatment, or having completed treatment. No sub‐groups analysis was completed on the impact of intervention timing, but the participants receiving the BMS intervention improved more than the CBT group on the function sections of the FACT and EORTC.

### 3.5 Social Well‐Being and Functioning (Table 4)

**TABLE 4:** Social function.

Author, year (level)	Prehab	Surgical post‐op acute	During or immediate post‐treatment	Survivor‐ship	Aerobic	Strength	Breathing	Other intervention	Societal integration impact (see key below)
Ferreira 2021 [90] (2)	X		X		X	X		Education, nutrition, address anxiety	NS SF‐36 social function (*p* = 0.091) (73.5 vs. 60), at 4 weeks
Huang 2019 [125] (2)	X		X					Web‐based education	NS EORTC‐QLQ‐C30 social function (P)
Bade 2021 [122] (2)			X		X			Education, relaxation, texting	NS EORTC‐QLQ‐C30 social function (S)
Hwang 2012 [126] (2)			X		X				NS EORTC‐QLQ‐C30 social
Quist 2018 [116] (2)			X		X	X		Health behavior counseling	NS EORTC‐QLQ‐C30 social function (S)
Ester 2021 [109] (3)			X		X	X		Education, nutrition	NS pre‐post leisure activity (S (feasibility))
Quist 2015 [121] (3)			X		X	X		Relaxation	Pre‐post decline FACT‐G social (S)
Quist 2020 [115] (2)			X		X	X		Relaxation	++ 1.2 FACT‐G social (S)
Sommer 2020 [133] (2)			X		X	X		Nutrition, smoking cessation, health behavior counseling	NS FACT‐G social
Park 2019 [114] (2)			X		X	X		Mobility app	**++ 8.2** EORTC‐QLQ‐C30 social function
Andersen 2013 [105] (3)			X		X		X		NS EORTC‐QLQ‐C30 30 social function (S)
Milbury 2019 [111] (2)			X					Yoga	+ 6.2 SF‐36 social function (S)
Raz 2016 [130] (4)			X					Supportive care sessions, psychological, social, spiritual	**++ 7.3** FACT‐G social (P)
Maddocks 2013 [124] (2)			X					Neuromuscular electrical stimulation	NS EORTC‐QLQ‐C30 social (S)
Schofield 2013 [132] (3)			X					Tailored supportive care, education	NS EORTC‐QLQ‐C30 social
Lau 2020 [127] (2)	X	X	X	?				Mind, body, spirit (including some physical exercise) vs. cognitive behavioral therapy	NS FACT‐G social (P/S unspecified)

*Note:* + = short‐term gain; ++ = long‐term gain; > = 4 weeks post intervention; bolded outcomes meet clinically meaningful change. Outcomes: EORTC‐QLQ‐C30 Q‐30 = European Organisation for Research and Treatment of Cancer‐Quality of Life Questionnaire‐Core 30; FACT‐G = Functional Assessment of Cancer Therapy‐General; SF‐36 = Short Form 36 Item Questionnaire.

Abbreviations: NS = non‐significant; P = primary; S = secondary.

**TABLE 5:** Key elements of all included studies are summarized.

Author, year (level)	Quality rating	Timing/setting				Treatment types			Functional outcome				Outcome type		Intervention				
		IP=inpatient; OP=outpatient; HB=homebased; X = not specified							1 = primary aim; 2 = secondary aim						S = supervised; MS = mixed supervision; US = unsupervised; NS = not specified; D = distant supervision				
		Prehab	Surg ical post op acute	During or immed iate POST‐Tx	Survivorship	Surgery	Non‐surgery	Palliative	Mobility	Social	Physical activity	General	Objective	PRO	Aerobic exercise	Strength training	Breathing exercise	Education	Other
Ahn 2021 (3)	Fair			OP		X	X		2			2	6MWT	EORTC QLQ C30	S	S			
Anderson 2013 (3)	Fair			OP, HB		X	X		1	1		1	ISWT or Yo‐yo (run test)	EORTC QLQ C30	MS		MS		
Arbane 2014 (2)	Fair		IP	HB		X			2		1	2	ISWT activity count	SF‐36	MS	MS			
Bade 2021 (2)	Good			HB			X	X		1	1	1	Step counts	EORTC QLQ C30; modifiable activity questionnaire	D			S	Relaxation, texting
Brocki 2016 (2)	Fair		IP			X			2				6MWT				S		
Brocki 2018 (2)	Good		IP			X			2		1		6MWT	EQ‐5D‐5L; PAS‐2			S		
Cheng 2020 (3)	Fair		IP			X			1				6MWT		NS	NS	NS		Nutrition
Dogan 2020 (2)	Good			OP			X		2				6MWT						Acupressure
Edbrooke 2019 (2)	Fair			HB			X		1		2		6MWT step counts	IPAQ	MS	MS			Behavior change strategies
Edvardsen 2015 (2)	Good			OP		X			2			2	Chair stands, stair runs, 1‐leg balance	SF‐36	S	S			
Ester 2021 (3)	Fair			OP, HP			X	X	2	2	2		6MWT; 1‐leg balance; 30sSTS	Leisure rating (GLTEQ, LSI); physical activity journal	MS	MS		Sleep, stress mgmt	Nutrition
Ferreira 2021 (2)	Good	HB		HB		X			1	2	2	2	6MWT	SF‐36; CHAMPS	US	US			Diet, anxiety
Finley 2021 (3)	Good	HB				X			1		1		6MWT, activity count		US				
Huang 2017 (2)	Good	IP				X			2			2	6MWT	EORTC QLQ C30	S	S	S		
Huang 2019 (2)	Fair	HB		HB			X			1		1		EORTC QLQ‐C30				US	Web‐based education
Hwang 2012 (2)	Good			OP		X	X			2		2		EORTC QLQ C30	S				
Jonsson 2019a, 2019b (2)	Fair, Good		IP	HB		X			1		1		6MWT; step counts	IPAQ‐E	S		S		
Kendall 2020 (3)	Poor			OP, HB		X			2		2	2	6MWT; PA counts	EuroQoL (EQ‐5D‐3L)			S		
Lafaro 2020 (3)	Fair	HB	IP	HB		X			2			2	6MWT; TUG; SPPB		US	US			
Lai Huang Yang 2017 (2)	Fair	IP				X			2			2	6MWT	EORTC QLQ C30	S		S		
Lai Su Qui 2017 (2)	Good	IP				X			2			2	6MWT	EORTC QLQ C30	S		S		
Lau 2020 (2)	Fair	OP		OP	Unclear	X	X				2	2		EORTC QLQ C30; FACT‐G					S I‐BMS and CBT
Li 2021 (2)	Good		IP			X			2				6MWT				US	US	
Licker 2017 (2); Bhatia 2019	Good	OP				X			2				6MWT		S	S			
Liu 2020 (2)	Good	HB				X			1			2	6MWT	WHODAS 2.0	MS	MS	MS	Relaxation	Whey protein supplement
Liu Feng 2022 (3)	Fair		IP			X			2				6MWT		S				
Liu Kuo 2021 (2)	Fair			HB		X			1				6MWT step counts		MS	MS	MS		
Ma 2021 (2)	Good	IP	IP			X			1				6MWT		S		S		
Maddocks 2013 (2)	Fair			IP				X		2	2	2	Step counts	EORTC QLQ C30					S NMES
Martinez‐Velilla 2021 (3)	Poor	Unclear				X	X					2		VES 13	MS	MS			MS‐Balance
McDonnell 2020 (3)	Fair				OP	Unclear	Unclear	Unclear	2				6MWT						S—Mindfulness based yoga
Messaggi‐Sartor 2019 (3)	Good			OP		X						2		EORTC QLQ C30	S	S	S		
Milbury 2019 (2)	Good			OP		X	X		1	2		2	6MWT	SF‐36					S Yoga
Minella 2021 (3)	Good	IP or HB				X			2				6MWT		MS	MS	MS		Nutrition psychological
Morano 2014 (2)	Fair	OP				X			2			2	6MWT UULEX	SF‐36	NS	NS		Stress mgmt, relaxation pre/post op care nutrition	Stretching
Naito 2019 (3) Tatemansu 2021 (3)	Good		HB	HB			X		1		1		5 m gait speed; 5XSTS step counts; PA 6MWT			MS			S Nutrition visits
Ozalevli 2010 (4)	Good			IP			X		2				6MWT		S	S	S		
Park 2019 (2)	Fair			HB			X	X	1	2		2	6MWT	EORTC QLQ C30	MS	MS			App
Pehlivan 2011 (2)	Fair	IP	IP			X			2				Walking distance, duration, speed		S		S		
Pehlivan 2019 (3)	Good	HB				X	X		1				6MWT		US		US		
Quist 2015 (3)	Fair			OP			X	X	2	2		2	6MWT	FACT‐G	S	S			Progressive relaxation
Quist 2018 (2)	Good			OP		X			2	2		2	6MWT	EORTC QLQ C30	S	S		S—Health promoting behaviors	S—Individual counseling
Quist 2020 (2)	Fair			OP			X	X	2	2		2	6MWT	FACT‐G	S	S			Relaxation
Raz 2016 (4)	Poor			OP		X				1		1		FACT‐G				Physical, psychological, social and spiritual needs	Interdisciplinary supportive care, team planning
Rutkowska 2019 (2)	Fair			IP			X	X	2				6MWT Fullerton Test		S	S	S		Relaxation
Salhi 2015 (2)	Good			X		X	X		1			2	6MWT	EORTC QLQ C30	S	S			S—WBVT
Schofield 2013 (3)	Fair			OP			X	X		2		2		EORTC QLQ C30				S—variable based on need (communication, emotional distress, sleeplessness, breathlessness, future goals)	
Sommer 2016 (2)	Fair	HB		OP		X			2				6MWT		MS	S	S	S—Health promotion	Counseling; nutrition
Sommer 2020 (2)	Good			OP		X				2		2		FACT‐G	S	S		Smoking cessation, nutrition	Counseling
Stigt 2013 (2)	Good			OP		X			2			2	6MWT	SF‐36	S	S			
Sui 2020 (2)	Good			HB		X						2		EORTC QLQ C30	US	US		US—App based health educ, exercise guidance	D—psychological support
Tenconi 2021 (2)	Fair	OP + HB	IP	OP + HB		X			1			2	6MWT	SF‐12	MS	MS	S		
Van der Leeden 2019 (3)	Fair	IP	IP			X			1			2	6MWT 30sSTS	SF‐36	S		US		
Yang 2018 (3)	Fair		IP			X			2				6MWT				S		Breathing ex with self‐efficacy enhancement

*Note:* Additional details can be found in Appendices [Link], [Link], [Link].

Abbreviations: 30sSTS, 30 s sit‐to‐stand; 5XSTS, 5 times sit‐to‐stand; 6MWT, six‐minute walk test; CBT, cognitive behavioral therapy; EORTC QLQ C30, European Organisation for Research and Treatment of Cancer Quality of Life Questionnaire Core 30; EQ‐5D‐5L, European Quality of Life 5 Dimensions 5 Level Version; FACT‐G, Functional Assessment of Cancer Therapy‐General; GLTEQ, Godin Leisure Time Exercise Questionnaire; I‐BMS, Integrative Mind Body Spirit; IPAQ, International Physical Activity Questionnaire; ISWT, Incremental Shuttle Walk Test; LSI, Leisure Score Index; NMES, neuromuscular electrical stimulation; PA, physical activity; PAS‐2, Patient Activity Scale II; SF‐12, 12 Item Short Form Survey; SF‐36, Short Form 36 Health Survey Questionnaire; SPPB, short physical performance battery; TUG, timed up and go; UULEX, Unsupported Upper Limb Exercise Test; VES‐13, Vulnerable Elders Survey; WBVT, whole‐body vibration therapy; WHODAS, World Health Organization Disability Assessment Schedule.

*Recommendations for Social Well‐Being and Functioning in the Prehabilitation Phase:*

- Insufficient evidence is available to make a recommendation for intervention to improve social function during Prehabilitation Phase.

A. Prehabilitation Phase

#### 3.5.1 Evidence Summary

While two studies [91, 126] included a prehabilitation phase for their intervention program, they were combined with ongoing interventions during treatment and thus do not allow for an understanding of the prehabilitation phase alone. In addition, neither of the studies found a significant effect on social function. Therefore, there is currently insufficient evidence that prehabilitation alone can impact social function outcomes.

*Recommendations for Social Well‐Being and Functioning in the During and Immediate Post‐Treatment Phase:*

- Dyadic (partner + patient) yoga may be used to improve social well‐being in the During and Immediate Post‐treatment Phase. (Grade C)
- Multimodal intervention including physical activity, nutritional support, and symptom management may be used to improve social function in the During and Immediate Post‐treatment Phase. (Grade C)

B. During and Immediate Post‐Treatment Phase

#### 3.5.2 Evidence Summary

##### 3.5.2.1 Aerobic Training (Grade B)

Two studies [123, 127] investigated the impact of aerobic exercise with some additional supportive care components in one of them. Bade et al. [122] compared an exercise and educational program to usual care in a cohort of people with advanced NSCLC. Hwang et al. [126] investigated the impact of high‐intensity aerobic exercise also in advanced lung cancer. Neither of these interventions significantly impacted social function outcomes.

##### 3.5.2.2 Combined Exercise Programs (Grade C)

Seven studies investigated the impact of combined exercise with other supportive care interventions on social function outcomes [106, 110, 115, 116, 117, 122, 134]. Six of these studies investigated the impact of combined aerobic and resistive exercise [110, 115, 116, 117, 122, 134], while one studied combined aerobic and breathing exercise [105]. Only two studies found statistically significant results in social function outcomes, with one reaching a clinically meaningful change [115, 116]. Park et al. [114] found an improvement in social function when a 12‐week app‐based aerobic and resistance training program was studied. Quist et al. [115] demonstrated significant improvement in social well‐being with a 12‐week supervised, structured exercise training program (aerobic, strength, and relaxation training) for people with advanced NSCLC. However, the change did not reach the clinically meaningful threshold. Ester et al. [109] and Sommer et al. [133] also investigated aerobic and resistance exercise programs of 12–14 weeks duration but did not find significant results. Thus, the impact of long‐term exercise programs on social function outcomes for lung cancer is currently unclear.

##### 3.5.2.3 Yoga (Grade C)

Milbury et al. [111] (Level 2 feasibility study) studied the impact of yoga training for patients and caregivers (dyadic Yoga) on physical and social as well as QOL. Patients were receiving thoracic radiation and had advanced NSCLC and dyads received yoga instruction two to three times/week for 6 weeks. Patients reported clinically meaningful improvement in physical and social well‐being. Caregivers reported marginally clinical improvement in role functioning and vitality.

##### 3.5.2.4 Supportive Care (Grade D)

In a quasi‐experimental design per Raz et al. [130], the intervention consisted of assessment of the patients and developing a care plan and educational sessions addressing multi‐domain needs of patients. The outcomes included FACT‐Lung and Lung Cancer Subscale (LCS) (symptom assessments). Social and family well‐being at 1 year was significantly better in the intervention as compared with the control group. However, a study by Schofield et al. [132] investigating the impact of a tailored supportive care program which reduced the number of unmet symptom needs, did not demonstrate a significant difference on social function.

**TABLE 6:** Summary of recommendations.

**Mobility**
*Prehabilitation phase*
Combined aerobic and other exercise (strengthening and/or breathing) should be used to improve mobility in the prehabilitation phase. (Grade B)
Breathing exercise should not be employed as the only type of exercise intervention to improve mobility in the prehabilitation phase. (Grade B)
*Surgical post‐operative acute phase*
Available evidence lacks consensus to make a recommendation for a combined exercise intervention to improve mobility during the surgical post‐operative acute phase
*During and immediate post‐treatment p*hase
A combined program of aerobic, resistance, and breathing exercise should be used to improve mobility in the During and Immediate Post‐treatment Phase. (Grade B)
**Physical activity**
*Prehabilitation phase*
Insufficient evidence is available to make a recommendation for interventions to improve physical activity during the Prehabilitation Phase
*Surgical post‐operative acute phase*
Combined intervention may be used to improve physical activity in the Surgical Post‐operative Acute Phase. (Grade C)
*During and immediate post treatment phase*
A multimodal program including aerobic exercise and education may be used to improve short‐term physical activity levels in individuals with late‐stage cancer. (Grade B)
**General function**
*Prehabilitation phase*
Insufficient evidence is available to make a recommendation for interventions to improve general function during the Prehabilitation Phase
*Surgical post‐operative acute phase*
Insufficient evidence is available for intervention effects on general function in the Surgical Post‐Operative Acute Phase.
*During and immediate post‐treatment phase*
Combined exercise programs including aerobic, strength, and breathing training with other supportive care interventions may be used to improve general function in the During and Immediate Post‐Treatment Phase. (Grade B)
Aerobic training alone may not be sufficient to improve self‐reported general function in the During and Immediate Post‐Treatment Phase. (Grade C)
**Social well‐being and functioning**
*Prehabilitation phase*
Insufficient evidence is available to make a recommendation for intervention to improve social function during Prehabilitation Phase
*During and immediate post‐treatment phase*
Dyadic (partner + patient) yoga may be used to improve social well‐being in the During and Immediate Post‐treatment Phase. (Grade C)
Combined intervention including physical activity, nutritional support, and symptom management may be used to improve social function in the During and Immediate Post‐treatment Phase. (Grade C)

## 4 Discussion

This CPG found the strongest support for recommending combined exercise interventions to improve functional outcomes including mobility and general function in persons with lung cancer. General physical activity has also been shown to be beneficial in improving function, but formal exercise interventions are more often studied. Combined programs typically consist of aerobic exercise combined with strengthening and/or breathing exercises, and sometimes include other multimodal strategies such as educational, symptom management, nutritional or psychological support measures. In this CPG, significant benefits of combined programs were found in both prehabilitation and during‐and‐immediate post‐treatment phases, with many studies finding clinically meaningful improvements relative to control interventions. As some studies examined only the prehabilitation phase, and others only the during‐and‐immediate post‐treatment phase, the relative impact of intervening preoperatively versus postoperatively remains indeterminate, as well as whether formally intervening in both phases is more advantageous than intervening in one phase or the other. Major takeaways include that (1) favorable findings are consistently seen for combined exercise in the prehabilitation phase toward mobility outcomes, which should be employed and (2) combined and/or multimodal exercise during the immediate post‐treatment phase is recommended toward all four of the outcome categories (mobility, physical activity, general function, and social function), including a “should” recommendation for mobility outcome goals during this phase.

While the focus of this literature is relatively narrow in that studies of patients undergoing surgical procedures predominate, intervention settings were heterogenous, ranging from supervised programs in hospitals, clinics, or research labs, to unsupervised or distantly supervised home programs, to mixed settings, suggesting these interventions can be administered in a variety of clinical or non‐clinical settings. Nonetheless, a sizeable number of studies were conducted in hospital or in hospital‐based clinics in part because of the convenience of enrolling patients and being able to deliver standard treatments. It is noteworthy that significant improvements were not limited to the supervised programs in this population, with some studies emphasizing home programs also demonstrating significant benefits on mobility outcomes [90, 95, 103], though beneficial findings were not universal in this regard [82, 91, 92, 121]. Technological integration, such as use of movement capture sensors, phone apps, and video sessions, emerged as a promising focus to boost clinical programs. One question that sometimes arises, with regards to systems of care in the United States, is whether exercise programs for lung cancer patients are most effectively delivered in cardiopulmonary rehabilitation settings with exercise specialists and pulmonologists, versus with qualified physical therapists and physiatrists in conventional rehabilitation settings. The current literature does not explicitly address this question.

Most studies appropriately note the substantial health challenges faced by patients with lung cancer, both related to the cancer and often to comorbidities, and they account for these issues in their inclusion and exclusion criteria, at varying levels. There remains a suboptimal understanding of how to approach patients at different health and performance levels regarding differential interventions and expected outcomes. Exercise studies do typically employ individualized physical exertion levels, often self‐rated based on tolerances, for the goal intensity level. A few studies have begun to build on this basic methodology, including individualized team‐based models [130], tiered intervention strategies [88, 136], inclusion of self‐modified back‐up interventions when patients are too ill for the regular program [77, 113], and subgroup analyses of participants at different performance levels [90]. Three studies, which were mindfulness and/or yoga interventions [112, 128, 136] incorporated a caregiver or partner into the intervention, and another, an acupressure study, allowed either self or caregiver performance of the intervention [106].

In general, the paucity of favorable findings with prehabilitation interventions regarding general function outcomes was surprising. Of the eleven studies of the impact of prehabilitation on general function, only two used an objective measure [89, 92]. Associations between objective and patient‐reported functional measures have received limited attention in the lung cancer population, with a recent retrospective study showing moderate‐level correlation between the objective measure of 30‐s sit‐to‐stand with a self‐reported physical function measure [138].

As noted previously, significant percentages of patients with Stages I and II disease do not receive surgery, even though it would be potentially curative, and the reasons presumably include the fact that many individuals are too unhealthy to undergo surgery. In this series, one paper found that patients originally deemed inoperable actually underwent surgery after a multimodal home‐based exercise intervention [89]. While most existing studies focus on optimizing surgical recovery and subsequent functional goals, the question of the role of rehabilitation in helping patients achieve operable, and potentially curative, status is an important question for future research.

For the survivorship phase category, the evidence became sparse on the impact of rehabilitative interventions to improve any form of function. Only one study includes substantial focus on long term survivors, a mindfulness and yoga trial with participants a mean 3–4 years post‐treatment [135]. While challenges likely exist in recruiting and controlling factors for intervention studies within the survivorship phase, evidence indicates high levels of functional deficits in this group, many of whom require homecare assistance, indicating the need for research in this area [139].

While our sample contains substantial literature inclusive of patients with advanced disease, either entirely or in mixed‐stage populations, conclusions remain difficult to draw based on heterogeneity of underlying contexts in this group which include patients undergoing chemotherapy [107, 113, 115, 118, 121, 122, 125], chemotherapy and/or radiation [106, 108, 114, 116, 123], chemotherapy and/or immunotherapy [109], targeted therapy [126], no/unknown oncologic treatment [123, 126, 128, 129], as well as varied interventions in the literature, including but not limited to exercise [106, 108, 110, 113, 114, 115, 118, 121, 122, 123, 126, 127, 129], mindfulness [127], neuromuscular electrical stimulation [124], acupressure [106], and needs assessment consultation [122]. While limited distinct conclusions can be drawn, multiple studies in this group of patients do show significant beneficial findings for mobility [107, 114, 115, 116, 118, 122], physical function [122], and general function [118, 128], supporting that individuals with advanced disease can be responsive to interventions promoting these outcomes. However, impact on social function was not seen in the few studies that examined this outcome category, therefore this outcome in particular would benefit from further study [110, 122, 123].

## 5 Strengths and Limitations

### 5.1 General

Limitations are present in the scope of the existing lung cancer rehabilitation literature itself, discussed below, and limitations also exist which relate to challenges inherent to conducting rehabilitation research. Future efforts will be needed to update these current guidelines as knowledge evolves. Regarding limitations inherent to rehabilitation interventional research, treatments tend to be global and multidimensional versus a precision approach. Wellness interventions such as nutrition, yoga, and/or psychological coping measures, are often lumped with exercise interventions in these studies and may especially lack precision. As a result, favorable findings can be ascertained, but causality of the individual components cannot be demonstrated. In particular, the ability to identify “should not” or “may not” intervention components may be especially challenging in the setting of mixed interventions, as was the case in this CPG, which generated only two such recommendations.

From phase and stage of disease standpoints, prehabilitation and near‐term post‐treatment care, especially for surgically managed patients, predominate. While some of this skew possibly relates to the prognostic and disease trajectory factors inherent with a lung cancer diagnosis, other factors likely apply as well, such as research feasibility often being most conducive surrounding the more intensive phases of oncological intervention. Additionally, the majority of the studies originated from Europe and Asia, possibly affecting the ability to be generalized to other national systems. Other differences in medical management of lung cancer may possibly occur between geographic regions that could confound the impact of rehabilitation interventions also impacting generalizability.

Most of the studies included safety observations, though degree of detail varied.

While limited medical harms were found, other types of harm are theoretically possible, such as financial burden of the interventions on the patient or health care system, and/or opportunity cost of time spent in performing the interventions rather than other valued life activities. On the other hand, it is also possible that the interventions may prove to have benefits in these regards. While our literature search was not geared toward these specific questions, they do merit further study.

### 5.2 Limitations of Interventions

Limitations are present in scope and quality of interventions assessed. Exercise is well represented in this literature, especially combined aerobic and resistance and/or breathing approaches. But few studies examine a particular type of exercise exclusively, such as aerobic, strengthening, or balance exercise, though some studies do attempt to determine the impact of breathing exercises. As noted above, a variety of treatment settings are represented in this literature. As to non‐exercise interventions, some literature exists about patient education on various topics, including, in some studies, the effect of employing remote or virtual methods for intervention or measurement, such as use of videos, apps, texting, phone calls, and activity trackers. But, many of these studies have not specifically been designed to investigate the effect of activity, an important gap that should be addressed going forward. While we found a few papers assessing caregiver dyad interventions, for the most part analysis of caregiver‐related aspects of functioning is lacking.

### 5.3 Limitations of Outcome Assessment

With regard to outcomes, mobility, physical activity, and general function have received greatest emphasis, with a lack of examination of self‐care or cognition, and with measurement of social functioning being essentially limited to self‐reported social subscales of global QOL instruments. Longer term outcomes, such as the survivorship phase, including employment also represent a gap area. More work is needed in strategies to sustain patient participation and motivation for effective interventions, including the possible role of supportive community organizations outside of primary medical care systems. Presence of more non‐cancer–related comorbidities has also been found to be a significant factor in reported functional limitations [140], a concern which is likely pertinent to lung cancer survivors.

A majority (72%) of the randomized controlled trials in the current sample have their functional measures as secondary outcomes, which could have negatively impacted the ability to capture significance of the interventions. Additionally, a few outcome measures predominate the data, especially 6MWT and self‐report QOL measures, which is a limitation regarding breadth of observations, but a strength in that use of the same measures facilitated comparison between different studies. Studies assessing function typically rely on proxy measures, such as the 6‐min walk test or self‐report tools, which correlate with function [141] but do not actually measure real world functioning.

Per our methods we aimed to generate recommendations when evidence graded at A, B, or C levels was available. Of note, while the majority of the studies assessed in this CPG were randomized trials (42 of 57 total studies, including 37 controlled studies rated as Level 2), no studies were determined to consist of Level 1 evidence. In short, the literature contained sufficient substrate to allow generation of multiple grade B and C recommendations, but no grade A recommendations. Furthermore, we encountered a few outcome subcategories for which the evidence, while containing individual studies consistent with B or C grades, was disparate between studies in either the nature of the interventions or in the significance of the findings. Additionally, when studies spanned multiple phases, the precise impact in time of the intervention could not always be determined. When a recommendation could not confidently be generated it was omitted. The disparate features of this body of literature, especially in treatment and post‐treatment phases, is a major limitation of this work.

### 5.4 Quality of the CPG

While we are an interdisciplinary group, comprised of two physical therapists and two physiatrists, other stakeholder groups are not represented in our core workgroup. Because our analysis was intentionally narrow to precisely target functional outcomes (and in the big picture we believe this is a strength of the present study), interesting and valuable findings that might have been obtained from casting a wider net were not captured. For example, we specifically did not include impairment‐level outcomes (i.e., strength, VO_2_ max, and pulmonary function tests), symptoms (fatigue, pain, and sleep), emotional or psychological factors, or hospitalization‐related parameters such as length of stay or medical complications, even though many studies examined these data points and often reported favorable findings. As a strength, we have defined a conceptual framework for potential future CPGs which generate function‐related recommendations, including for evaluating other types of cancer. This framework, consisting of rigorous definitions surrounding function, including domains of assessment and relevant outcome measures, were defined and honed over the course of this effort. While future efforts will require their own unique aspects of decision‐making, we believe that the methodology presented in this current guideline can be readily adapted to future function‐related evidence‐based guidelines, which are needed to advance patient care in oncology rehabilitation.

## 6 Conclusions

The literature on patient function in lung cancer emphasizes mobility outcomes, in the context of prehabilitation and early post‐treatment phases, with moderate level demonstrable benefits of combined exercise programs. Areas where further study is needed, and which future updates of this guideline should consider, include critical components of exercise programs, potential differences in effectiveness for patient subgroups, and sustainability of performing the interventions. There is also a need for examination of an increased breadth of interventions and functional outcome domains, as well as further exploration of specific contexts including advanced disease, survivorship, high medical complexity and frailty, and caregiver‐related factors. Longer term studies looking at community based functional outcomes assessing participation and societal integration are needed.

## Author Contributions

**Mary Vargo:** conceptualization (equal), formal analysis (equal), investigation (equal), methodology (equal), validation (equal), visualization (equal), writing – original draft (equal), writing – review and editing (equal). **Lynn H. Gerber:** conceptualization (equal), formal analysis (equal), investigation (equal), methodology (equal), resources (equal), validation (equal), visualization (equal), writing – original draft (equal), writing – review and editing (equal). **Laura S. Gilchrist:** conceptualization (equal), formal analysis (equal), investigation (equal), methodology (equal), validation (equal), visualization (equal), writing – original draft (equal), writing – review and editing (equal). **Mary Insana Fisher:** conceptualization (equal), formal analysis (equal), investigation (equal), methodology (equal), project administration (lead), resources (equal), validation (equal), visualization (equal), writing – original draft (equal), writing – review and editing (equal).

## Conflicts of Interest

The authors declare no conflicts of interest.

## Supporting information


Appendix S1.

Appendix S2.

Appendix S3.

Appendix S4.

Appendix S5.

Appendix S6.

Appendix S7.

Appendix S8.

Appendix S9.

## Data Availability

The data is included in the appendices.
