# Supplementary material for: Recommendations for Interventions to Improve Function in Patients With Lung Cancer: A Clinical Practice Guideline
Source: Cancer Med. 2025 Jul 4;14(13):e70626. doi: 10.1002/cam4.70626 (PMC12231241; doi:10.1002/cam4.70626)
Supplement: Supplementary file 1 — Appendix S1. [file CAM4-14-e70626-s007.docx]

**Appendix A – MEDLINE Search**

| (((("Lung Neoplasms"[Mesh] OR neoplasm[tiab] OR neoplasms[tiab] OR neoplasms[mesh] OR cancer[tiab] OR cancers[tiab] OR carcinoma[tiab] OR carcinomas[tiab] OR sarcoma[tiab] OR sarcomas[tiab] OR lymphoma[tiab] OR lymphomas[tiab] OR glioma[tiab] OR gliomas[tiab] OR melanoma[tiab] OR melanomas[tiab] OR neoplasia[tiab] OR neoplasias[tiab] OR malignant[tiab] OR malignancy[tiab] OR malignancies[tiab] OR leukemia[tiab] OR leukemias[tiab] OR "Leukemia"[Mesh] OR sarcoma[mesh] OR lymphoma[mesh] OR glioma[mesh] OR melanoma[mesh] OR malignant tumor[tiab] OR malignant tumour[tiab] OR chemotherapy[tiab] OR chemotherapies[tiab] OR radiotherapy[tiab] OR radiotherapies[tiab] OR "radiation therapy"[tiab] OR "radiation therapies"[tiab] OR immunotherapy[tiab] OR immunotherapies[tiab] OR oncology[tiab] OR "radiation treatment"[tiab] OR "radiation treatments"[tiab] OR "Radiotherapy"[Mesh] OR "hormonal therapy"[tiab] OR "hormonal therapies"[tiab] OR "immunological therapy"[tiab] OR "immunological therapies"[tiab] OR "Immunotherapy"[Mesh] OR "Estrogen Replacement Therapy"[Mesh] OR "Molecular Targeted Therapy"[Mesh] OR "targeted therapy"[tiab] OR "Hematologic Neoplasms"[Mesh] OR "Hematopoietic Stem Cell Transplantation"[Mesh] OR "Hematopoietic stem cell transplant"[tiab] OR "Hematopoietic stem cell transplants"[tiab] OR "Hematopoietic stem cell transplantation"[tiab] OR "Hematopoietic stem cell transplantations"[tiab])) AND ("Lung"[Mesh] OR "lungs"[tiab] OR "lung"[tiab] OR "Pulmonary"[tiab])) AND ((rehabilitation[tiab] OR rehabilitation[mh] OR rehabilitation[sh] OR prehabilitation[tiab] OR physiotherapy[tiab] OR physiotherapies[tiab] OR physiatry[tiab] OR physiatrist[tiab] OR physiatrists[tiab] OR "physical therapy"[tiab] OR "physical therapies"[tiab] OR "physical therapy modalities"[mh] OR "exercise therapy"[tiab] OR "exercise therapies"[tiab] OR "exercise therapy"[mh] OR "cognitive therapy"[tiab] OR "cognitive therapies"[tiab] OR "Cognitive Behavioral Therapy"[Mesh] OR "occupational therapy"[tiab] OR "occupational therapies"[tiab] OR "occupational therapy"[mh] OR "speech therapy"[tiab] OR "speech therapies"[tiab] OR "speech therapy"[mh] OR "psychosocial intervention"[tiab] OR "psychosocial interventions"[tiab] OR psychoeducation[tiab] OR "social support"[tiab] OR "Social Support"[mesh] OR "Rehabilitation, Vocational"[Mesh] OR "vocational rehabilitation"[tiab] OR neuropsychology[tiab] OR neuropsychologies[tiab] OR "physical activity"[tiab] OR "physical activities"[tiab]))) AND ("Recovery of Function"[Mesh] OR Function[tiab] OR functional[tiab] OR functions[tiab] OR functioning[tiab] OR impairment[tiab] OR impairments[tiab] OR "dysfunction"[tiab] OR "dysfunctions"[tiab] OR "dysfunctional"[tiab] OR fatigue[tiab] OR fatigues[tiab] OR fatigued[tiab] OR fatiguing[tiab] OR "Mental Fatigue"[Mesh] OR "Fatigue"[Mesh] OR exhaustion[tiab] OR exhausted[tiab] OR exhausting[tiab] OR exhausts[tiab] OR tired[tiab] OR tiredness[tiab] OR tiring[tiab] OR "low alertness"[tiab] OR pain[tiab] OR "Pain"[Mesh] OR "Cancer Pain"[Mesh] OR pains[tiab] OR painful[tiab] OR distress[tiab] OR ache[tiab] OR aches[tiab] OR aching[tiab] OR ached[tiab] OR neuropathy[tiab] OR neuropathies[tiab] OR neuropathic[tiab] OR arthralgia[tiab] OR arthralgias[tiab] OR "Arthralgia"[Mesh] OR somatic[tiab] OR "post surgical"[tiab] OR "Cognitive Decline"[tiab] OR "cognitive declines"[tiab] OR "Cognitive Dysfunction"[Mesh] OR "Mild Neurocognitive Disorder"[tiab] OR "Mild Neurocognitive Disorders"[tiab] OR "Mental Deterioration"[tiab] OR "Mental Deteriorations"[tiab] OR cognition[tiab] OR "functional capacities"[tiab] OR "Chronic Limitation of Activity"[tiab] OR "Activities of Daily Living"[Mesh] OR "daily living activity"[tiab] OR "daily living activities"[tiab] OR "activities of daily living"[tiab] OR "activity daily living"[tiab] OR "Self Care"[Mesh] OR "self-care"[tiab] OR "selfcare"[tiab] OR ambulation[tiab] OR ambulat*[tiab] OR dress[tiab] OR dressing[tiab] OR bathing[tiab] OR washing[tiab] OR IADL[tiab] OR "instrumental activity daily living"[tiab] OR "Self-Management"[Mesh] OR "self management"[tiab] OR "selfmanagement"[tiab] OR "Memory"[Mesh] OR memory[tiab] OR memories[tiab] OR recall[tiab] OR recalls[tiab] OR "processing speed"[tiab] OR "processing speeds"[tiab] OR chemobrain[tiab] OR "chemo brain"[tiab] OR chemofog[tiab] OR "chemo fog"[tiab] OR "cancer related cognitive changes"[tiab] OR "cancer related cognitive decline"[tiab] OR "cancer related cognitive deficits"[tiab] OR neuropsychological[tiab] OR neuropsych[tiab] OR "Language"[Mesh] OR language[tiab] OR speech[tiab] OR learning[tiab] OR "Learning"[Mesh] OR "speech intelligibility"[tiab] OR "Speech Intelligibility"[Mesh] OR "Speech Intelligibilities"[tiab] OR "mouth opening"[tiab] OR "mouth openings"[tiab] OR trismus[tiab] "Trismus"[Mesh] OR lockjaw[tiab] OR "communication disorder"[tiab] OR "communication disorders"[tiab] OR "Deglutition"[Mesh] OR deglutition[tiab] OR swallowing[tiab] OR "Deglutition Disorders"[Mesh] OR "swallowing disorder"[tiab] OR "swallowing disorders"[tiab] OR Dysphagia*[tiab] OR "oral intake"[tiab] OR "tube feeding"[tiab] OR "Enteral Nutrition"[Mesh] OR "Speech, Esophageal"[Mesh] OR "esophageal speech"[tiab] OR "esophageal voice"[tiab] OR "esophageal voices"[tiab] OR "oesophageal voice"[tiab] OR "oesophageal voices"[tiab] OR "Larynx, Artificial"[Mesh] OR "artificial larynx"[tiab] OR "Voice Prosthesis"[tiab] OR "voice prostheses"[tiab] OR "Laryngeal Prosthesis"[tiab] OR "laryngeal prostheses"[tiab] OR "tracheoesophageal voice"[tiab] OR "tracheoesophageal voices"[tiab] OR "tracheoesophageal prosthesis"[tiab] OR "tracheoesophageal prostheses"[tiab] OR "Quality of Life"[Mesh] OR "quality of life"[tiab] OR "life quality" OR HRQOL[tiab] OR QOL[tiab] OR "Shortness of Breath"[tiab] OR breathlessness[tiab] OR breathless[tiab] OR "Dyspnea"[Mesh] OR dyspnea[tiab] OR dyspneas[tiab] OR "labored breathing"[tiab] OR "labored breathes"[tiab] OR "difficulty breathing"[tiab] OR "Breath Shortness"[tiab] OR "breath shortnesses"[tiab] OR "Return to Work"[Mesh] OR "return to work"[tiab] OR employment[tiab] OR "back to work"[tiab] OR "Sexual Dysfunction, Physiological"[Mesh] OR "Physiological Sexual Disorder"[tiab] OR "Physiological Sexual Disorders"[tiab] OR "Sexual Dysfunctions, Psychological"[Mesh] OR "fecal incontinence"[tiab] OR "Bowel Incontinence"[tiab] OR "Fecal Incontinence"[Mesh] OR "urinary incontinence"[tiab] OR "Urinary Incontinence"[Mesh] OR dyspareunia[tiab] OR "Dyspareunia"[Mesh] OR "genital pain"[tiab] OR "genital pains"[tiab] OR vulvodynia[tiab] OR vulvodynias[tiab] OR Vestibulodynia[tiab] OR Vestibulodynias[tiab] OR "Vulvodynia"[Mesh] OR "pelvic pain"[tiab] OR "Pelvic Pain"[Mesh] OR "Male Impotence"[tiab] OR "Erectile Dysfunction"[Mesh] OR "Pelvic Floor Disorder"[tiab] OR "pelvic floor disorders"[tiab] OR "Pelvic Floor Disorders"[Mesh] OR sexuality[tiab] OR Sexuality[mesh] OR "physical functioning"[tiab] OR "physical function"[tiab] OR "physical functions"[tiab] OR "physical fitness"[tiab] OR "Physical Fitness"[Mesh] OR exercise[tiab] OR exercise[mesh] OR exercising[tiab] OR exercises[tiab] OR exercised[tiab] OR mobility[tiab] OR mobile[tiab] OR "Mobility Limitation"[Mesh] OR ambulation[tiab] OR ambulat*[tiab] OR "difficulty walking"[tiab] OR "difficulties walking"[tiab] OR "mobility limitation"[tiab] OR "mobility limitations"[tiab] OR "Physical Endurance"[Mesh] OR endurance[tiab] OR "physical endurance"[tiab] OR stamina[tiab] OR "Range of Motion, Articular"[Mesh] OR "range of motion"[tiab] OR "joint flexibility"[tiab] OR "joint flexibilities"[tiab] OR flexibility[tiab] OR flexible[tiab] OR "Pliability"[Mesh] OR pliability[tiab] OR pliable[tiab] OR "Gait"[Mesh] OR gait[tiab] OR gaits[tiab] OR balance[tiab] OR "Postural Balance"[Mesh] OR "Bone Density"[Mesh] OR "bone density"[tiab] OR "bone densities"[tiab] OR "Muscle Strength"[Mesh] OR "muscle strength"[tiab] OR ((strength*[tiab] OR tone[tiab]) AND muscl*) OR "Body Weight"[Mesh] OR "body weight"[tiab] OR "body weights"[tiab] OR "body composition"[tiab] OR "Body Composition"[Mesh] OR "body compositions"[tiab] OR "Position Senses"[tiab] OR "Sense of Position"[tiab] OR "Proprioception"[Mesh] OR Proprioception[tiab] OR Proprioceptions[tiab] OR "Sensory Function"[tiab] OR "sensory functions"[tiab] OR "Sensation"[Mesh] OR sensation[tiab] OR sensations[tiab] OR sensory[tiab] OR sensories[tiab] OR "Sarcopenia"[Mesh] OR sarcopenia[tiab] OR sarcopenias[tiab] OR "Accidental Falls"[Mesh] OR fall[tiab] OR falls[tiab] OR falling[tiab] OR dexterity[tiab] OR "fine motor"[tiab] OR "vestibular function"[tiab] OR "vestibular functions"[tiab] OR "vestibular functioning"[tiab] OR vestibular[tiab] OR "lean mass"[tiab] OR myopathy[tiab] OR myopathies[tiab] OR myopathic[tiab] OR ataxia[tiab] OR "Ataxia"[Mesh] OR dizziness[tiab] OR "Light-Headedness"[tiab] OR Lightheadedness[tiab] OR "Dizziness"[Mesh] OR "Visual Acuity"[Mesh] OR "visual acuity"[tiab] OR "visual acuities"[tiab] OR vision[tiab] OR bone[tiab] OR bones[tiab] OR "venous thrombic event"[tiab] OR "tumor lysis syndrome"[tiab] OR "cerebellar degeneration"[tiab] OR "graft vs host disease"[tiab] OR "superior vena cava syndrome"[tiab] OR "radiation fibrosis"[tiab] OR "scar tissue"[tiab] OR "scar adhesion"[tiab] OR "oncologic emergency"[tiab] OR "oncologic emergencies"[tiab]) |
| --- |
