# Supplementary material for: Recommendations for Interventions to Improve Function in Patients With Lung Cancer: A Clinical Practice Guideline
Source: Cancer Med. 2025 Jul 4;14(13):e70626. doi: 10.1002/cam4.70626 (PMC12231241; doi:10.1002/cam4.70626)
Supplement: Supplementary file 2 — Appendix S2. [file CAM4-14-e70626-s001.docx]

**Appendix B – Minimal Clinically Important Differences for Selected Outcome Measures**

| **Outcome Measure** | **MCID** | **Reference Population** |
| --- | --- | --- |
| 30-second Chair Stand | Not Reported | NA |
| 6 Minute Walk Test | 30 m | Lung Cancer^70^ |
| Accelerometry/Daily Step Count | 600 steps/day | COPD^74,76^ |
| Daily Activity | 11 min | COPD, arterial disease^68,69^ |
| EORTC-QLQ-C30 Physical Function | 6 | Multiple Cancers SR^142^ |
| EORTC-QLQ-C30 Role Function | 12 | Multiple Cancers SR^142^ |
| EORTC-QLQ-C30 Social Function* | 6 (MID) | Multiple Cancers |
| FACT Functional Wellbeing | 2 | Multiple Cancers^143^ |
| FACT-L Social | Not Reported | NA |
| FACT-TOI | 5 | Lung Cancer^144^ |
| FACT-L | 2 | Lung Cancer^144^ |
| Fullerton | Not Reported | NA |
| Maximal Walking Distance | Not Reported | NA |
| SF-36 | 2 | Pulmonary Fibrosis^145^ |
| SF-36 Physical Function | 10 | Chronic Lung Disease^146^ |
| SF-36 Social | 12.5 | Chronic Lung Disease^146^ |
| SPPB | 1 | Older adults^73^ |

EORTC-QLQ-C30 = European Organisation for Research and Treatment of Cancer, Quality of Life Questionnaire, Core 30

FACT = Functional Assessment of Cancer Therapy

FACT-TOI = Functional Assessment of Cancer Therapy, Total Outcome Index

FACT-L = Functional Assessment of Cancer Therapy, Lung

SF-36 = 36-Item Short Form Survey

SBBP = Short Physical Performance Battery

* Musoro et al. EORTC Melanoma, Breast, Head and Neck, Genito-urinary, Gynecological, Gastro-intestinal, Brain, Lung and Quality of Life Groups. Minimally important differences for interpreting EORTC QLQ-C30 change scores over time: A synthesis across 21 clinical trials involving nine different cancer types. Eur J Cancer. 2023 Jul;188:171-182. doi: 10.1016/j.ejca.2023.04.027. Epub 2023 May 7. PMID: 37257278.
