# Supplementary material for: Recommendations for Interventions to Improve Function in Patients With Lung Cancer: A Clinical Practice Guideline
Source: Cancer Med. 2025 Jul 4;14(13):e70626. doi: 10.1002/cam4.70626 (PMC12231241; doi:10.1002/cam4.70626)
Supplement: Supplementary file 6 — Appendix S6. [file CAM4-14-e70626-s005.docx]

| **National Institutes of Health Bias Assessment - Controlled Trials** | | | | | | | | | | | | | | | |
| --- | --- | --- | --- | --- | --- | --- | --- | --- | --- | --- | --- | --- | --- | --- | --- |
| **Author, Year** | **Overall Rating** | **Q1 Random-ized Trial** | **Q2 Adequate Random-**  **ization** | **Q3 Treatment allocation** | **Q4 Treatment Blinded** | **Q5 Assessor Blinded** | **Q6 Baseline Similar** | **Q7 Dropout Rate <20%** | **Q8 Dropout Rate Diff <15%** | **Q9 High Adherence (>70%)** | **Q10 Other Intervent-**  **ions Avoided** | **Q11 Valid Reliable Outcomes** | **Q12 Sample Size**  **Adeq** | **Q13 Analysis Prespecif** | **Q14**  **ITT** |
| **Arbane, 2014** | Fair | Yes | Yes | Yes | No | No | No | No | Yes | Unclear | Yes | Yes | Yes | Yes | Unclear |
| **Bade, 2021** | Good | Yes | Yes | Yes | No | Unclear | Yes | Yes | Yes | No | Yes | Yes | Yes | Yes | Yes |
| **Bhatia, 2019** | Good | Yes | Yes | Yes | No | Yes | Yes | Yes | Yes | Yes | Yes | Yes | Unclear | Yes | Yes |
| **Brocki, 2016** | Fair | Yes | Yes | Yes | No | Yes | Yes | Yes | Yes | Yes | Unclear | Yes | Yes | Unclear | Yes |
| **Brocki, 2018** | Fair | Yes | Yes | Yes | No | Yes | Yes | Yes | Yes | Not Reported | Yes | Yes | No | Yes | Yes |
| **Cheng, 2020** | Fair | Yes | Unclear | Not Reported | No | Not Reported | Yes | Yes | Yes | Not Reported | Yes | Yes | No | Yes | Yes |
| **Dogan, 2020** | Good | Yes | Yes | Yes | No | Yes | Yes | No | Yes | Unclear | Yes | Yes | Yes | Yes | No |
| **Edvardsen, 2015** | Good | Yes | Yes | Yes | No | Yes | Yes | Yes | Yes | Yes | Yes | Yes | Yes | Yes | Yes |
| **Edbrooke, 2019** | Fair | Yes | Yes | Yes | No | Yes | Unclear | Yes | Yes | No | Yes | Yes | Yes | Yes | Yes |
| **Ferreira, 2021** | Good | Yes | Yes | Yes | No | Yes | Yes | Yes | Yes | Yes | Unclear | Yes | Yes | Yes | Yes |
| **Huang, 2017** | Good | Yes | Yes | Yes | No | Yes | Yes | Yes | Yes | Not Reported | Yes | Yes | Yes | Yes | Yes |
| **Huang, 2019** | Fair | Yes | Yes | Unclear | Unclear | Unclear | Yes | Unclear | Unclear | Unclear | Yes | Yes | Yes | Yes | Unclear |
| **Hwang, 2012** | Fair | Yes | Yes | Yes | No | Yes | Yes | No | No | Yes | Yes | Yes | Unclear | Yes | Yes |
| **Jonsson, 2019a** | Fair | Yes | Yes | Yes | No | Yes | Yes | Yes | Yes | Not Reported | Yes | Yes | No | Yes | Unclear |
| **Jonsson, 2019b** | Good | Yes | Yes | Yes | No | Yes | Yes | Yes | Yes | Yes | Yes | Yes | No | Yes | Yes |
| **Kendall, 2020** | Poor | Yes | Yes | Unclear | Unclear | Unclear | Yes | No | No | Unclear | No | Yes | Unclear | Yes | No |
| **Lai, Huang, Yang, 2017** | Fair | Yes | Yes | Not Reported | No | Yes | Yes | Yes | No | Not Reported | Yes | Yes | Yes | Yes | Yes |
| **Lai, Su, Qui, 2017** | Good | Yes | Yes | Yes | No | Yes | Yes | Yes | Yes | Yes | Yes | Yes | Yes | Yes | Yes |
| **Lau, 2020** | Fair | Yes | Yes | Unclear | Yes | Yes | Yes | No | Yes | Unclear | Unclear | Yes | No | Not Reported | Yes |
| **Li, 2021** | Good | Yes | Yes | Yes | No | Yes | Yes | Yes | Yes | Yes | Yes | No | Yes | Not Reported | Yes |
| **Licker, 2016** | Good | Yes | Yes | Yes | No | Yes | Yes | Yes | Yes | Yes | Yes | Yes | Yes | Yes | Yes |
| **Liu, 2020** | Good | Yes | Yes | Unclear | No | Yes | Yes | Yes | Yes | Unclear | Yes | Yes | Yes | Not Reported | Yes |
| **Liu, 2021** | Fair | Yes | Yes | Yes | No | Yes | Yes | Yes | Yes | Unclear | Unclear | Yes | Yes | Yes | No |
| **Liu, 2022** | Fair | No | No | No | No | No | Yes | Yes | Yes | NA | No | Yes | No | Yes | Unclear |
| **Ma, 2021** | Good | Yes | Yes | Yes | No | Yes | Yes | Yes | Yes | Not Reported | Yes | Yes | Yes | Yes | Not Reported |
| **Maddocks, 2013** | Fair | Yes | Yes | Unclear | No | No | Yes | No | Yes | No | Not Reported | Yes | Yes | Yes | No |
| **Martinez-Velilla, 2021** | Poor | No | No | No | No | No | Yes | Yes | Yes | Unclear | Yes | Not Reported | Unclear | Yes | Yes |
| **Messaggi-Sartor, 2019** | Good | Yes | Yes | Yes | No | Yes | Yes | No | Yes | Yes | Yes | Yes | Yes | Yes | Yes |
| **Milbury, 2019** | Good | Yes | Yes | Yes | No | Yes | Yes | Yes | Yes | Yes | Yes | Yes | Yes | Yes | Yes |
| **Morano, 2014** | Fair | Yes | Yes | Yes | No | Not Reported | Yes | Yes | Yes | Not Reported | Yes | Yes | Not Reported | Yes | Yes |
| **Pehlivan, 2011** | Fair | Yes | No | Yes | No | Not Reported | Yes | Not Reported |  | Not Reported | Not Reported | Yes | Not Reported | Yes | No |
| **Quist, 2018** | Good | Yes | Yes | Yes | No | Yes | Yes | No | Yes | Not Reported | Yes | Yes | Yes | Yes | Yes |
| **Quist, 2020** | Fair | Yes | Unclear | Unclear | Unclear | Yes | Yes | No | Yes | No | Yes | Yes | Yes | Yes | Yes |
| **Rutkowska, 2019** | Fair | Yes | Yes | Unclear | Unclear | Unclear | Yes | Yes | Yes | Unclear | Yes | Yes | Yes | Yes | No |
| **Salhi, 2015** | Good | Yes | Yes | Yes | No | No | Yes | Yes | Yes | Unclear | Yes | Yes | Yes | Yes | Yes |
| **Schofield, 2013** | Fair | Yes | Yes | Yes | No | No | Yes | No | Yes | Yes | Not Reported | Yes | No | Yes | No |
| **Sommer, 2016** | Fair | Yes | Yes | Yes | No | Not Reported | Unclear | No | Yes | No | Yes | Yes | No | Yes | Yes |
| **Sommer, 2020** | Good | Yes | Yes | Yes | No | Yes | Yes | No | Yes | Not Reported | Yes | Yes | Yes | Yes | Yes |
| **Stigt, 2013** | Good | Yes | Yes | Yes | No | No | Yes | No | No | No | Unclear | Yes | Yes | Yes | Yes |
| **Sui, 2020** | Good | Yes | Yes | Yes | No | Not Reported | Yes | Yes | Yes | Not Reported | Yes | Yes | Yes | Yes | Yes |
| **Tenconi, 2021** | Fair | Yes | Yes | Yes | No | No | Unclear | No | Yes | Yes | Yes | Yes | Yes | Yes | Yes |
| **Yang, 2018** | Fair | No | No | Unclear | Yes | Yes | Yes | Yes | Yes | Not Reported | Not Reported | Yes | Yes | Yes | No |
| **Bias Assessment Questions** | | | | | | | | | | | | | | | |
| 1. Was the study described as randomized, a randomized trial, a randomized clinical trial, or an RCT? | | | | | | | | | | | | | | | |
| 2. Was the method of randomization adequate (i.e., use of randomly generated assignment)? | | | | | | | | | | | | | | | |
| 3. Was the treatment allocation concealed (so that assignments could not be predicted)? | | | | | | | | | | | | | | | |
| 4. Were study participants and providers blinded to treatment group assignment? | | | | | | | | | | | | | | | |
| 5. Were the people assessing the outcomes blinded to the participants' group assignments? | | | | | | | | | | | | | | | |
| 6. Were the groups similar at baseline on important characteristics that could affect outcomes (e.g., demographics, risk factors, co-morbid conditions)? | | | | | | | | | | | | | | | |
| 7. Was the overall drop-out rate from the study at endpoint 20% or lower of the number allocated to treatment? | | | | | | | | | | | | | | | |
| 8. Was the differential drop-out rate (between treatment groups) at endpoint 15 percentage points or lower? | | | | | | | | | | | | | | | |
| 9. Was there high adherence to the intervention protocols for each treatment group? | | | | | | | | | | | | | | | |
| 10. Were other interventions avoided or similar in the groups (e.g., similar background treatments)? | | | | | | | | | | | | | | | |
| 11. Were outcomes assessed using valid and reliable measures, implemented consistently across all study participants? | | | | | | | | | | | | | | | |
| 12. Did the authors report that the sample size was sufficiently large to be able to detect a difference in the main outcome between groups with at least 80% power? | | | | | | | | | | | | | | | |
| 13. Were outcomes reported or subgroups analyzed prespecified (i.e., identified before analyses were conducted)? | | | | | | | | | | | | | | | |
| 14. Were all randomized participants analyzed in the group to which they were originally assigned, i.e., did they use an intention-to-treat analysis? | | | | | | | | | | | | | | | |
