# Supplementary material for: Recommendations for Interventions to Improve Function in Patients With Lung Cancer: A Clinical Practice Guideline
Source: Cancer Med. 2025 Jul 4;14(13):e70626. doi: 10.1002/cam4.70626 (PMC12231241; doi:10.1002/cam4.70626)
Supplement: Supplementary file 7 — Appendix S7. [file CAM4-14-e70626-s009.docx]

|  | **National Institutes of Health Bias Assessment - Pre/post-test Study Design** | | | | | | | | | | | | |
| --- | --- | --- | --- | --- | --- | --- | --- | --- | --- | --- | --- | --- | --- |
| **Author, Year** | **Overall Rating** | **Q1  Objective Clear** | **Q2  Eligibility Criteria** | **Q3 Representative Sample** | **Q4  Prespecified Eligibility** | **Q5  Sample Size** | **Q6  Intervention Description** | **Q7  Outcome Valid Reliable** | **Q8  Assessors Blinded** | **Q9  Loss of Follow-up <20%** | **Q10  Stats on Change in Outcome** | **Q11  Multiple assessments pre and post** | **Q12  Group Level Stats** |
| **Ahn, 2021** | Fair | Yes | Yes | Yes | Not Reported | Not Reported | Yes | Yes | No | No | Yes | No | NA |
| **Andersen, 2013** | Fair | Yes | Yes | Not Reported | Yes | Not Reported | Yes | Yes | Not Reported | No | Yes | No | No |
| **Ester, 2021** | Fair | Yes | Yes | Unclear | No | No | Yes | Yes | NA | No | Yes | No | NA |
| **Finley, 2021** | Fair | Yes | Yes | Yes | Yes | No | Yes | Yes | No | No | Yes | No | NA |
| **Lafaro, 2020** | Fair | Yes | No | Yes | No | Yes | Yes | Yes | Unclear | No | Yes | Yes | NA |
| **McDonnel, 2020** | Fair | Yes | Yes | Yes | No | No | Yes | Yes | Unclear | Yes | Yes | No | NA |
| **Minella, 2021** | Good | Yes | Yes | Yes | No | Unclear | Yes | Yes | No | Yes | Yes | No | NA |
| **Naito, 2019** | Good | Yes | Yes | Yes | No | Yes | Yes | Yes | Not Reported | Yes | Yes | Yes | NA |
| **Park, 2019** | Fair | Yes | Yes | Yes | Unclear | Yes | Yes | Yes | Unclear | Yes | Yes | No | No |
| **Pehlivan, 2019** | Good | Yes | Yes | Yes | Yes | Yes | Yes | Yes | Not Reported | No | Yes | No | NA |
| **Quist, 2015** | Fair | Yes | Yes | Yes | No | Unclear | Yes | Yes | No | No | Yes | No | Yes |
| **Tatemansu, 2021** | Good | Yes | Yes | Yes | No | Yes | Yes | Yes | Not Reported | Yes | Yes | Yes | NA |
|  |  |  |  |  |  |  |  |  |  |  |  |  |  |
|  |  |  |  |  |  |  |  |  |  |  |  |  |  |
|  |  |  |  |  |  |  |  |  |  |  |  |  |  |
|  | **Bias Assessment Questions** | |  |  |  |  |  |  |  |  |  |  |  |
|  | 1. Was the study question or objective clearly stated? | | | | | | | | | | |  |  |
|  | 2. Were eligibility/selection criteria for the study population prespecified and clearly described? | | | | | | | | | | |  |  |
|  | 3. Were the participants in the study representative of those who would be eligible for the test/service/intervention in the general or clinical population of interest? | | | | | | | | | | |  |  |
|  | 4. Were all eligible participants that met the prespecified entry criteria enrolled? | | | | | | | | | | |  |  |
|  | 5. Was the sample size sufficiently large to provide confidence in the findings? | | | | | | | | | | |  |  |
|  | 6. Was the test/service/intervention clearly described and delivered consistently across the study population? | | | | | | | | | | |  |  |
|  | 7. Were the outcome measures prespecified, clearly defined, valid, reliable, and assessed consistently across all study participants? | | | | | | | | | | |  |  |
|  | 8. Were the people assessing the outcomes blinded to the participants' exposures/interventions? | | | | | | | | | | |  |  |
|  | 9. Was the loss to follow-up after baseline 20% or less? Were those lost to follow-up accounted for in the analysis? | | | | | | | | | | |  |  |
|  | 10. Did the statistical methods examine changes in outcome measures from before to after the intervention? Were statistical tests done that provided p values for the pre-to-post changes? | | | | | | | | | | |  |  |
|  | 11. Were outcome measures of interest taken multiple times before the intervention and multiple times after the intervention (i.e., did they use an interrupted time-series design)? | | | | | | | | | | |  |  |
|  | 12. If the intervention was conducted at a group level (e.g., a whole hospital, a community, etc.) did the statistical analysis take into account the use of individual-level data to determine effects at the group level? | | | | | | | | | | |  |  |
|  |  | | | | | | | | | | |  |  |
