# Supplementary material for: Recommendations for Interventions to Improve Function in Patients With Lung Cancer: A Clinical Practice Guideline
Source: Cancer Med. 2025 Jul 4;14(13):e70626. doi: 10.1002/cam4.70626 (PMC12231241; doi:10.1002/cam4.70626)
Supplement: Supplementary file 9 — Appendix S9. [file CAM4-14-e70626-s006.docx]

|  | **National Institutes of Health Bias Assessment - Case Series** | | | | | | | | | |
| --- | --- | --- | --- | --- | --- | --- | --- | --- | --- | --- |
| **Author, Year** | **Overall Rating** | **Q1 Objective Clear** | **Q2 Population Defined** | **Q3 Consecutive Cases** | **Q4 Subjects Comparable** | **Q5 Interventions Described** | **Q6 Outcomes Valid Reliable** | **Q7 Follow Up Adequate** | **Q8 Stats Described** | **Q9 Results Described** |
| **Ozalevi, 2010** | Good | Yes | Yes | Unclear | No | Yes | Yes | Yes | Yes | Yes |
|  |  |  |  |  |  |  |  |  |  |  |
|  |  |  |  |  |  |  |  |  |  |  |
|  | **Bias Assessment Questions** | | |  |  |  |  |  |  |  |
|  | 1. Was the study question or objective clearly stated? | | | | | | | |  |  |
|  | 2. Was the study population clearly and fully described, including a case definition? | | | | | | | |  |  |
|  | 3. Were the cases consecutive? | | | | | | | |  |  |
|  | 4. Were the subjects comparable? | | | | | | | |  |  |
|  | 5. Was the intervention clearly described? | | | | | | | |  |  |
|  | 6. Were the outcome measures clearly defined, valid, reliable, and implemented consistently across all study participants? | | | | | | | |  |  |
|  | 7. Was the length of follow-up adequate? | | | | | | | |  |  |
|  | 8. Were the statistical methods well-described? | | | | | | | |  |  |
|  | 9. Were the results well-described? | | | | | | | |  |  |
